# d^10^s^2^ Post-Transition Metal Anions: Identifying and Analyzing Their Dual-Mode Lewis Basicity

**DOI:** 10.1021/acs.jpclett.4c03649

**Published:** 2025-03-10

**Authors:** Jake M. Seymour, Ekaterina Gousseva, Lewis G. Parker, Frances K. Towers Tompkins, Richard M. Fogarty, Lennart Frankemoelle, Rebecca Rowe, Coby J. Clarke, David A. Duncan, Robert G. Palgrave, Roger A. Bennett, Patricia A. Hunt, Kevin R. J. Lovelock

**Affiliations:** † Department of Chemistry, University of Reading, Reading RG6 6DX, U.K.; ‡ Department of Materials, Imperial College London, London SW7 2AZ, U.K.; § Department of Chemistry, Imperial College London, London SW7 2AZ, U.K.; ∥ School of Chemistry, University of Nottingham, Nottingham NG7 2RD, U.K.; ⊥ Diamond Light Source, Didcot, Oxfordshire OX11 0DE, U.K.; # Department of Chemistry, University College London, London WC1H 0AJ, U.K.; g School of Chemical and Physical Sciences, Victoria University of Wellington, Wellington 6012, New Zealand

## Abstract

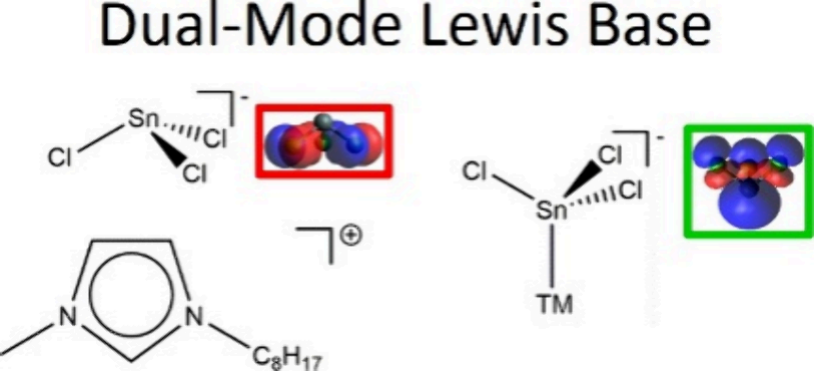

Liquid-phase d^10^s^2^ post-transition metal anions, such as [SnCl_3_]^−^, appear in a range of applications with a focus on catalysis and material preparation. However, little is known about their electronic structure and how it relates to reactivity. Using X-ray photoelectron spectroscopy and *ab initio* calculations, we demonstrate that liquid-phase d^10^s^2^ post-transition metal anions can act as dual-mode Lewis bases, interacting through the metal center and/or the ligands, with the interaction mode depending on the identity of the Lewis acid/electron acceptor. The Lewis basicity of the metal donor atom is controlled mainly by the metal identity; the ligand can be used for fine-tuning. Changing the metal center has a strong effect on the ligand basicity. These findings provide insight into d^10^s^2^ post-transition metal anion electronic structure, which will enable better molecular-level design of catalytic systems.

d^10^s^2^ post-transition metal anions often have a stereochemically active lone pair, e.g., the trigonal-pyramidal trihalostannate anions [Sn^II^Cl_3_]^−^ and [Sn^II^Br_3_]^−^ or halobismuthate anions,^1,2^ that gives rise to unusual liquid-phase electronic properties and reactivity.^1−5^ They have potential uses in materials chemistry, such as optoelectronic applications^2,6^ and preparation and disposal/recycling of semiconductor materials employed within devices (energy storage, electronic, and optical),^7−11^ and are present in halide perovskites (although the structures tend to be different).^12−14^ Furthermore, [SnCl_3_]^−^ has been extensively used as a ligand in liquid-phase catalytic processes due to the ability of [SnCl_3_]^−^ to act as a Lewis base (i.e., electron donor) toward a (Lewis acidic) transition metal center (i.e., electron acceptor) to form Sn–transition metal bonds and thus tune catalytic properties.^4,5,15−21^ [SnCl_3_]^−^ Lewis basic activity occurs in both molecular liquids^4,5,15−18^ and ionic liquids (ILs);^19−21^ e.g., Pt–SnCl_3_ complexes have been used for hydroformylation reactions.^21^ The presence of the lone pair on Sn(II) is crucial to the formation of the transition metal–Sn bond.^4,5,15,17,18,22^ [SnCl_3_]^−^ has also been used as a liquid-phase catalyst in its own right in the absence of any transition metals.^23,24^

The electronic structures of liquid-phase d^10^s^2^ anions are relatively underexplored, despite the unusual and attractive properties, because they are spectroscopically quiet; e.g., UV–vis spectroscopy rarely provides insight. Advances have been driven mainly by empirical and iterative synthetic experimentation. The low volatility of ILs^1,25−28^ means X-ray photoelectron spectroscopy (XPS) of liquid-phase halometallate anions in ILs offers opportunities to characterize their electronic structure. Therefore, there is the prospect of an improved understanding of metal and ligand combinations to underpin the choice of metal complex for catalysis.

There are apparent contradictions in the electron donor ability of d^10^s^2^ halometallate anions for the two existing electron donor scales. An XPS-derived anion electrostatic interaction strength scale for 39 anions, including three d^10^s^2^ post-transition metal halometallate anions ([SnCl_3_]^−^, [SnBr_3_]^−^, and [Bi_2_Cl_8_]^2–^), has been measured using XPS electron binding energies of cationic nitrogen, *E*_B_(N_cation_ 1s), for [C_8_C_1_Im][A] ILs, where [C_8_C_1_Im]^+^ (1-octyl-3-methyldialkylimidazolium) acts as an electron acceptor (Lewis acid) probe.^26,28−31^ The order of anion electrostatic interaction strength suggests that [SnCl_3_]^−^ is a midranking electron donor: Cl^–^ ≈ Br^–^ > [ZnCl_4_]^2–^ ≈ [ZnBr_4_]^2–^ > [Bi_2_Cl_8_]^2–^ ≈ [SnCl_3_]^−^ ≈ [CF_3_SO_3_]^−^ > [InCl_4_]^−^ ≈ [InBr_4_]^−^.^26,28,29^ In contrast, experimental ionization energies, *E*_i_ (where a large *E*_i_ equates to poor electron donor ability^32^), suggest that [SnCl_3_]^−^ is a very strong electron donor: [SnCl_3_]^−^ ≈ Br^–^ < Cl^–^ ≈ [ZnBr_4_]^2–^ < [ZnCl_4_]^2–^ < [CF_3_SO_3_]^−^ < [InCl_4_]^−^.^27^ Clearly, the orders of anion *E*_i_(27) and anion electrostatic interaction strength^28^ are not the same; Cl^–^ and [ZnCl_4_]^2–^ have greater electrostatic interaction strengths than [SnCl_3_]^−^, but *E*_i_ is smaller for [SnCl_3_]^−^ than Cl^–^ and [ZnCl_4_]^2–^.

In this work, we use valence and core XPS, supported by calculations, to characterize the electronic structure of d^10^s^2^ and d^10^s^0^ post-transition metal anions (two different metals and three different anionic ligands). This allows us to explain the apparent contradiction of the *E*_i_ and electrostatic interaction strength scales for post-transition metal anions. Furthermore, we explain and predict the electron donor modes for the post-transition metal anion with respect to two different types of Lewis acid, organic cations, and other metal cations (e.g., Pt). Note that the terms electron donor and Lewis base are often used interchangeably; a Lewis base has the ability to provide a pair of electrons to coordinate to a Lewis acid.^33^ Therefore, our demonstration of the electron donor modes for post-transition metal anions is equivalent to the Lewis basicity of the post-transition metal anions.

Our new valence XPS and calculation results show that the highest occupied MO (HOMO^34^) of formally d^10^s^2^ [SnCl_3_]^−^ has strong Sn 5s + Sn 5p contributions. Valence XPS of [C_8_C_1_Im][SnCl_3_] showed a peak due to the HOMO at electron binding energy *E*_B_(HOMO) = *E*_i_ = 3.1 eV (Figure 1a), at an *E*_B_ lower than that for free Cl^–^ at *E*_B_(HOMO) = 3.5 eV (Figure 2b,e). The fitted component at *E*_B_ = 3.1 eV increased in area by 14 times relative to the component at *E*_B_ = 4.8 eV when *hν* was increased from 250 to 6000 eV (Figure 1a and Table S14). On the basis of XPS experiments and atomic orbital (AO) photoionization cross sections, the HOMO peak at 3.1 eV can be identified as arising from a molecular orbital (MO^34^) with strong Sn contributions. The computationally derived Gelius-weighted density of states (DoS) showed the same trend (Figure 1c) that experimental XPS did (Figure 1a), validating the calculations. Calculations on [SnCl_3_]^−^ determine a single antibonding MO as the HOMO with a Mulliken population analysis showing the following contributions: Sn 5s, ∼30%; Sn 5p, ∼20%; and Cl 3p, ∼50% (Figure 1b and Table S10). These results match literature calculations.^15,17,22^ In contrast, the experimental peak at *E*_B_ = 4.8 eV (Figure 1a) has strong Cl 3p AO contributions with minimal Sn 5s + 5p AO contributions and matches very well to the [SnCl_3_]^−^ calculated HOMO–1 to HOMO–5, which as Mulliken population analysis shows contain >97% Cl 3p contributions (Table S10).

**Figure 1:**
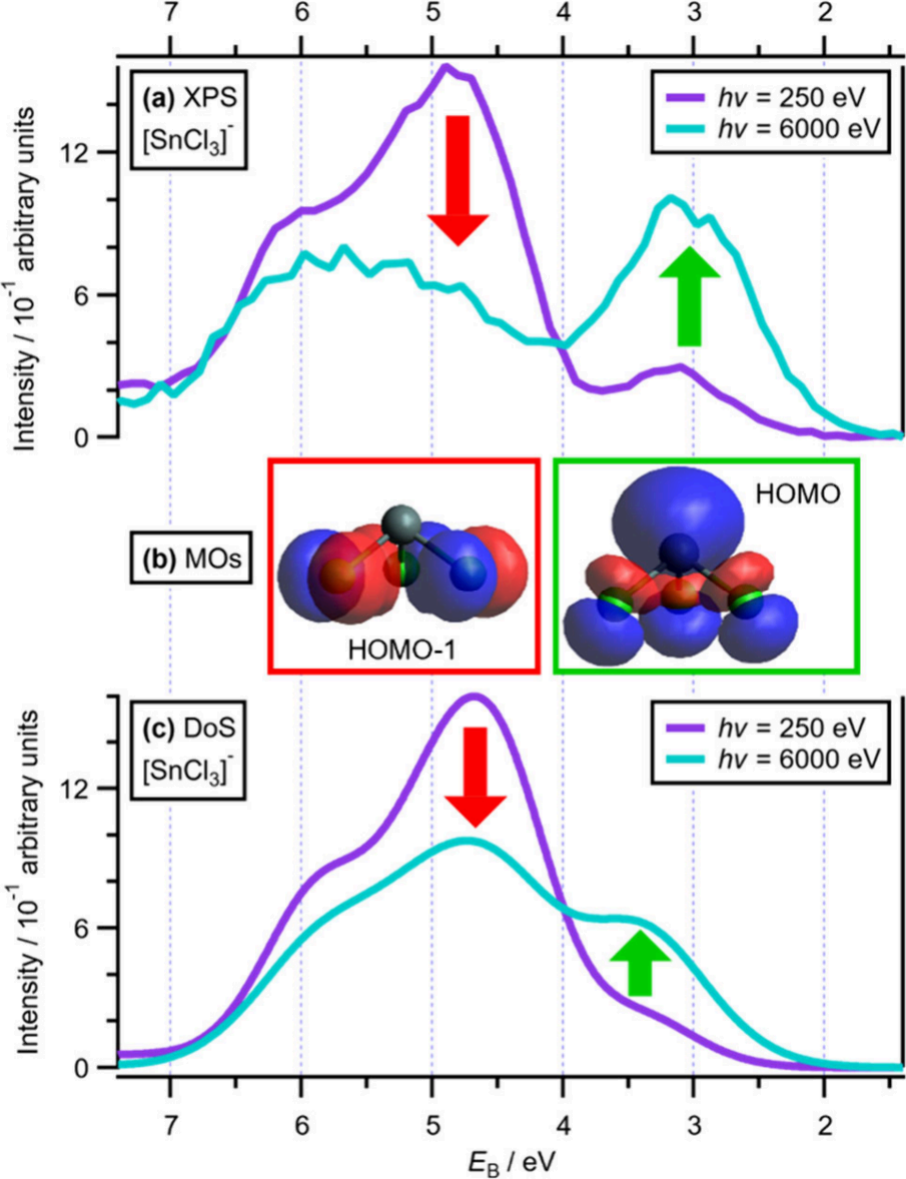
(a) Area-normalized experimental valence XP spectra for [C_8_C_1_Im][SnCl_3_] at *hν* values of 250 and 6000 eV. (b) Representative MOs of the two major groups of molecular orbitals for [SnCl_3_]^−^. The box colors match the arrow colors in panels a and c. (c) Area-normalized Gelius-weighted DoS calculations for a sum of [C_8_C_1_Im]^+^ and [SnCl_3_]^−^ at *hν* = 250 eV and [SnCl_3_]^−^ at *hν* = 6000 eV. The areas were normalized using procedures outlined in section 8 of the Supporting Information. All XP spectra were charge referenced to *E*_B_(C_alkyl_ 1s) = 285.00 eV.^27^ Further details about the procedures used for charge referencing of XP spectra are outlined in section 6 of the Supporting Information. The arrows indicate the change in peak intensity going from *hν* = 250 eV to *hν* = 6000 eV.

**Figure 2:**
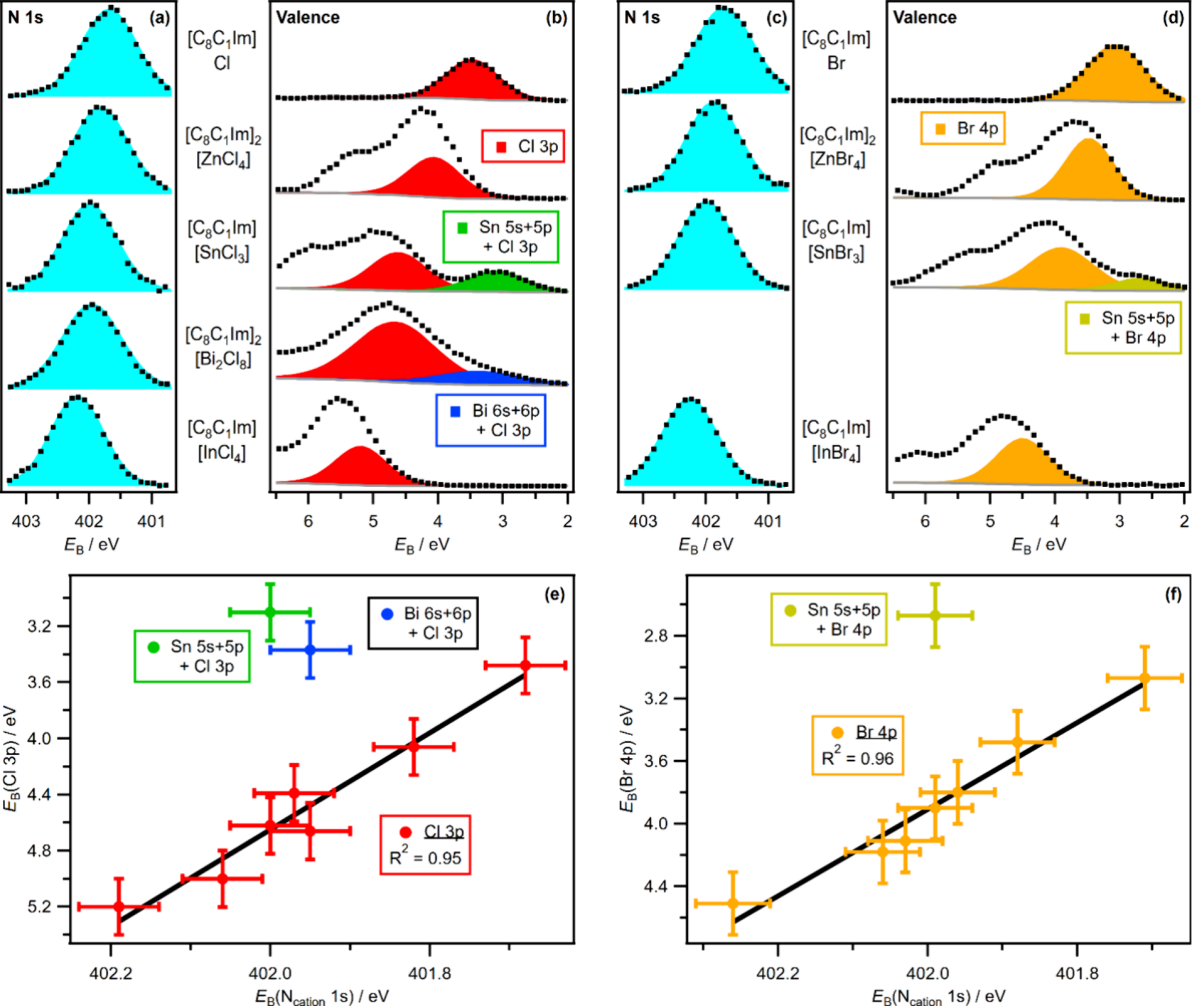
Visual comparison of area-normalized XP spectra recorded at *hν* = 1486.6 eV, vertically offset for the sake of clarity: (a) N_cation_ 1s for five Cl-containing ILs, (b) valence for five Cl-containing ILs, with Cl 3p antibonding fitted components for the four metal complex ILs, the Cl 3p fitted component for [C_8_C_1_Im]Cl, and the anion HOMO for [C_8_C_1_Im][SnCl_3_], (c) N_cation_ 1s for four Br-containing ILs, and (d) valence for four Br-containing ILs, with Br 4p antibonding fitted components for the three metal complex ILs, the Br 4p fitted component for [C_8_C_1_Im]Br, and the anion HOMO for [C_8_C_1_Im][SnBr_3_]. Further details about the procedures used for charge referencing of XP spectra are outlined in section 6 of the Supporting Information. The areas of the XP spectra were normalized using procedures outlined in section 8 of the Supporting Information. XPS electron binding energy, *E*_B_, correlations: (e) *E*_B_(Cl 3p) vs *E*_B_(N_cation_ 1s) for seven Cl-containing ILs and (f) *E*_B_(Br 4p) vs *E*_B_(N_cation_ 1s) for seven Br-containing ILs.

We predict that the HOMO for any [Sn(anion)_3_]^−^ anion will have strong Sn 5s + Sn 5p contributions, whether the anion is Cl^–^, Br^–^, [CF_3_SO_3_]^−^, or other anions popular in ILs, e.g., [N(CF_3_SO_2_)_2_]^−^ {bis[(trifluoromethane)sulfonyl]imide}. The HOMO for [SnBr_3_]^−^, giving a peak at *E*_B_ = 2.7 eV (Figure 2d), has strong Sn 5s + Sn 5p AO contributions, as the experimental valence XPS for [SnBr_3_]^−^ gives a shape very similar to that of [SnCl_3_]^−^, with a relatively small peak at a low *E*_B_ due to the HOMO with strong Sn contributions (panels b and d, respectively, of Figure 2). The *E*_B_(HOMO) of 2.7 eV for [SnBr_3_]^−^ (Figure 2d,f) is lower than the *E*_B_(HOMO) for free Br^–^ at *E*_B_ = 3.1 eV (Figure 2d,f). We conclude, on the basis of data for both [SnCl_3_]^−^ and [SnBr_3_]^−^, that for an ionic liquid containing a metal cation, if we observe a peak at an *E*_B_ lower than the *E*_B_ for the free anion, there must be a relatively strong metal cation contribution to the HOMO. Therefore, we can conclude that the HOMO for [Sn(CF_3_SO_3_)_3_]^−^ has strong Sn 5s + Sn 5p contributions, as demonstrated by *E*_B_(HOMO) ∼ 4.0 eV for [Sn(CF_3_SO_3_)_3_]^−^ (Figure S19) being lower than *E*_B_(HOMO) = 5.0 eV for free [CF_3_SO_3_]^−^ (Figure S19 and ref (35)).

The HOMO for the [Bi_2_Cl_8_]^2–^ anion has an observable Bi contribution. Valence XPS of [C_8_C_1_Im]_2_[Bi_2_Cl_8_] gave a relatively low intensity shoulder at *E*_i_ ∼ 3.4 eV (Figure 2b and Figures S16 and S17), matching the Sn-based anions but differing from the d^10^s^0^ anions, e.g., [C_8_C_1_Im]_2_[ZnCl_4_] (Figure 2b and Figures S16 and S17). Furthermore, for DFT calculations of [Bi_2_Cl_8_]^2–^, the HOMO and HOMO–1 (as there are two Bi centers in the complex) have 9% Bi 6s and 88% Cl 3p contributions [from a Mulliken population analysis (Table S15)]; the MO representation also shows a clear Bi contribution (Table S15). There are far smaller contributions to the HOMO by Bi 6s in halobismuthate anions than by Sn 5s + Sn 5p in [SnCl_3_]^−^ (Figure 2b and Tables S10 and S13). These differences in both AO contributions to the HOMO and *E*_i_ can be explained by the difference in energy between the metal *n*s valence AO and the Cl 3p valence AO Δ*E*_B_(M *n*s × Cl 3p). For [SnCl_3_]^−^, the small Δ*E*_B_(M *n*s – Cl 3p) (relative to [Bi_2_Cl_8_]^2–^) allowed better overlap between Sn 5s and Cl 3p, relative to Bi 6s and Cl 3p, thus explaining the differences in both HOMO and *E*_i_. The peak at *E*_B_ ∼ 3.1 eV (Figure 2b and Figures S16 and S17) was due to Cl 3p contributions, matching very well to the [Bi_2_Cl_8_]^2–^ HOMO–2 to HOMO–17 with >98% Cl 3p contributions [from a Mulliken population analysis (Table S15)].

A very close match of the valence electronic structure was observed for the [SnCl_3_]^−^ anion (a discrete anion in the IL) to three perovskites with [cation][SnCl_3_] stoichiometry (see Figure S12 for a direct visual comparison of the data).^13^ Solid-state [cation][SnCl_3_] perovskites, despite having the same chlorostannate stoichiometry as the liquid-phase [SnCl_3_]^−^ anion, have a very different local structure around the Sn atom; Sn is surrounded by six Cl atoms with no stereochemically active lone pair in the perovskite, unlike in the molecular [SnCl_3_]^−^ anion. This finding suggests that the electronic structure for the [SnCl_3_]^−^ anion is independent of the anion structure; i.e., it is not closely related to the trigonal pyramidal geometric structure for the [SnCl_3_]^−^ anion.

For the formally d^10^s^0^ metal chlorometallate complexes [ZnCl_4_]^2–^ and [InCl_4_]^−^, the HOMOs are composed of Cl 3p AOs with minimal metal *n*s or *n*p contributions (Figure 2b and Tabe S15), in clear contrast to [SnCl_3_]^−^ and [Bi_2_Cl_8_]^2–^. This finding is demonstrated by the excellent visual match of the experimental and calculated valence XPS for [ZnCl_4_]^2–^ and [InCl_4_]^−^ (Figure S18). For both [ZnCl_4_]^2–^ and [InCl_4_]^−^, the HOMO to HOMO–7 all had >99% Cl 3p contributions [from a Mulliken population analysis (Table S15)]. A more detailed breakdown of the nature of the MOs matched to experimental XPS peaks is given in Figures S16 and S17.

We predict that halobismuthate anions would be weaker Lewis bases (i.e., electron donors) to (Lewis acidic) transition metals (i.e., electron acceptors) through the anion HOMO than [SnCl_3_]^−^, giving weaker Bi–transition metal bonds than Sn–transition metal bonds. This prediction is based upon (i) the significantly weaker contribution of the Bi 6s for the HOMO of halobismuthate anions relative to that of Sn 5s for the HOMO of [SnCl_3_]^−^ and (ii) the *E*_i_ for [Bi_2_Cl_8_]^2–^ being larger than that for [SnCl_3_]^−^. Our prediction matches the literature evidence, which shows plentiful examples of transition metal–[SnCl_3_]^−^ bonds,^4,5,15−21^ but no reports of transition metal halobismuthate anion bonds.^36^ Furthermore, we predict that for [PbCl_3_]^−^, another formally d^10^s^2^ post-transition metal halometallate anion,^3^ the HOMO will have Pb 6s character somewhere between those of [SnCl_3_]^−^ and [Bi_2_Cl_8_]^2–^, e.g., 20–30% Pb 6s contribution, as *E*_B_(Pb 6s) is between *E*_B_(Sn 5s) and *E*_B_(Bi 6s).^37^ Therefore, we predict that [PbCl_3_]^−^ would be a weaker Lewis base to (Lewis acidic) transition metals through the anion HOMO than [SnCl_3_]^−^ but stronger than [Bi_2_Cl_8_]^2–^.

The ability of [Sn(anion)_3_]^−^ anions to donate electron density through the Sn center can be tuned using the anionic ligand identity and readily judged using core XPS data. On the basis of core-level XPS data, [SnCl_3_]^−^ and [SnBr_3_]^−^ will be better electron donors through the Sn center than [Sn(CF_3_SO_3_)_3_]^−^. Via core-level XPS, *E*_B_(Sn 3d) and *E*_B_(Sn 4d) are significantly smaller, by ∼1.2 eV, for [Sn(CF_3_SO_3_)_3_]^−^ than for [SnCl_3_]^−^ and [SnBr_3_]^−^ (Figure S20). Therefore, we can conclude that there is a lower electron density on the Sn center for [Sn(CF_3_SO_3_)_3_]^−^ than for [SnCl_3_]^−^ and [SnBr_3_]^−^. We predict that [Sn(CH_3_CO_2_)_3_]^−^ will be a good electron donor through the Sn center, as the electron donor ability of [CH_3_CO_2_]^−^ is similar to or even better than those of Cl^–^ and Br^–^.^28,30^ Conversely, using a weakly electron donating anion {e.g., [N(CF_3_SO_2_)_2_]^−^ or [(C_2_F_5_)_3_PF_3_]^−^^28^} will make the Sn center a weaker electron donor. These findings complement those found for neutral bismuth electron donors, in which switching from halide ligands to more electron donating ligands is expected to increase the electron density at Bi and make Bi–transition metal bonds more favorable.^36^

The observed trend in the anion *E*_i_ ([SnCl_3_]^−^ < [Bi_2_Cl_8_]^2–^ ≈ Cl^–^ < [ZnCl_4_]^2–^ < [InCl_4_]^−^) can be readily explained using the results presented so far. The trend in *E*_i_ (Cl^–^ < [ZnCl_4_]^2–^ < [InCl_4_]^−^) exists because the bonding of Cl^–^ to a metal center increases *E*_B_ for MOs dominated by Cl 3p contributions, i.e., the HOMO for [ZnCl_4_]^2–^ and [InCl_4_]^−^. The trend in *E*_i_ ([SnCl_3_]^−^ < [Bi_2_Cl_8_]^2–^ ≈ Cl^–^) can be readily explained by the metal s and p contributions to the HOMO. The observed trend in the anion *E*_i_ ([SnBr_3_]^−^ < Br^–^ < [ZnBr_4_]^2–^ < [InBr_4_]^−^) can be explained by the same factors, as can the observed trend in the anion *E*_i_ {[Sn(CF_3_SO_3_)_3_]^−^ < [CF_3_SO_3_]^−^}.

For halometallate anions, the anion electrostatic interaction strength scale [measured using the *E*_B_(N_cation_ 1s) of organic cation [C_8_C_1_Im]^+^^26,28,29^] is determined by the ability of the halide ligands (attached to the metal center) to donate electron density to [C_8_C_1_Im]^+^. For the chlorometallate ILs and [C_8_C_1_Im]Cl, there are excellent visual (Figure 2a,b) and linear (Figure 2e; *R*^2^ = 0.95) correlations between *E*_B_(Cl 3p) (i.e., MOs with strong Cl 3p contributions) and *E*_B_(N_cation_ 1s). Furthermore, for the bromometallate ILs and [C_8_C_1_Im]Br, there are excellent visual (Figure 2c,d) and linear (Figure 2f; *R*^2^ = 0.96) correlations between *E*_B_(Br 4p) (i.e., MOs with strong Br 4p contributions) and *E*_B_(N_cation_ 1s). Overall, as *E*_B_(halide *n*p) increases so does *E*_B_(N_cation_ 1s) (Figure 2), demonstrating that a larger *E*_B_(halide *n*p) corresponds to weaker anion electrostatic interaction on the scale measured using organic cation [C_8_C_1_Im]^+^.^26,28,29^ As differences in *E*_B_(N_cation_ 1s) are caused by differences in the anion–cation nonspecific electrostatic interactions,^28^ the halide–[C_8_C_1_Im]^+^ interactions are best explained as nonspecific electrostatic interactions (i.e., charge-controlled interactions). Therefore, the donation of electrons for halometallate anions to [C_8_C_1_Im]^+^ is primarily driven by halide–[C_8_C_1_Im]^+^ interactions and not by metal–[C_8_C_1_Im]^+^ interactions (Figure 3).

**Figure 3:**
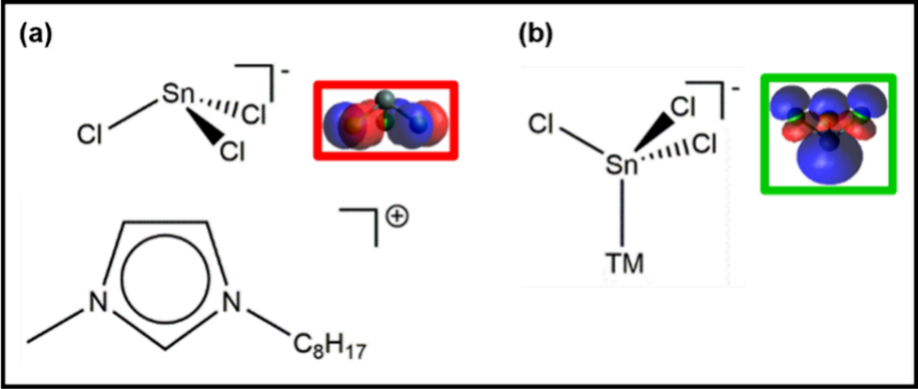
Interaction mode for dual-mode Lewis base anion [SnCl_3_]^−^. (a) Cl ligands acting as a Lewis base with respect to an organic Lewis acid, along with a representation of the HOMO in the same orientation. (b) Sn center acting as a Lewis base with respect to a transition metal center (i.e., Lewis acid), along with a representation of HOMO–1 in the same orientation.

[SnCl_3_]^−^ and [SnBr_3_]^−^ can be described as dual-mode Lewis bases [i.e., electron donors (Figure 3)]. First, [SnCl_3_]^−^ and [SnBr_3_]^−^ can donate electron density to organic cation Lewis acids through the halide atoms, as shown here. Second, [SnCl_3_]^−^ and [SnBr_3_]^−^ can also donate electron density to transition metal Lewis acids through the Sn atom, as demonstrated by the occurrence of transition metal–Sn bonds.^18^ Both [Sn(CF_3_SO_3_)_3_]^−^ and [Bi_2_Cl_8_]^2–^ could also act as dual-mode Lewis bases, although they can do more weakly through the metal centers than [SnCl_3_]^−^ and [SnBr_3_]^−^. Conversely, [InCl_4_]^−^, [ZnCl_4_]^2–^, Cl^–^, [InBr_4_]^−^, [ZnBr_4_]^2–^, and Br^–^ are single-mode Lewis bases, as these anions can donate electron density through only the halide atoms (Figure 3).

Once [SnCl_3_]^−^ has formed a Sn–transition metal bond, the SnCl_3_ moiety retains an electron density distribution similar to that of free [SnCl_3_]^−^ anions;^17^ the SnCl_3_ moiety retains the ability to act also as a Lewis base through the Cl atoms. Therefore, [SnCl_3_]^−^ has the potential to act as a dual-mode Lewis base for applications such as catalysis, as has been achieved for other species.^38^ Furthermore, varying the organic/inorganic countercation could lead to different SnCl_3_–countercation interactions, which could lead to altered transition metal–SnCl_3_ interactions, giving further tunability of catalysis; it has already been shown that changing the organic cation can affect catalysis using Pt–SnCl_3_ complexes.^20^

We have identified and characterized the dual-mode Lewis basicity of liquid-phase d^10^s^2^ post-transition metal anions, demonstrating the importance of both the formal lone pair on the metal center and the ligand identity. For future work, there are exciting opportunities to use liquid jet XPS to probe the electronic structure of d^10^s^2^ metal anions, given that both transition metal complexes and non-aqueous solvents have already been studied successfully.^39,40^

## Data Availability

The data underlying this study are openly available in the University of Reading Research Data Archive at https://doi.org/10.17864/1947.001407.

## Supporting Information Available

Additional experimental details, data analysis methods, and XPS and DFT results, including a demonstration of sample purity (PDF)
