# Supplementary material for: d10s2 Post-Transition Metal Anions: Identifying and Analyzing Their Dual-Mode Lewis Basicity
Source: J Phys Chem Lett. 2025 Mar 10;16(11):2831–6. doi: 10.1021/acs.jpclett.4c03649 (PMC11931533; doi:10.1021/acs.jpclett.4c03649)
Supplement: Supplementary file 1 — jz4c03649_si_001.pdf [file jz4c03649_si_001.pdf]

Supplementary Information

$d^{10}s^2$ Post-Transition Metal Anions: Identifying and Analyzing their Dual-Mode Lewis Basicity

Jake M. Seymour,[a] Ekaterina Gousseva,[a] Lewis G. Parker,[a] Frances K. Towers Tompkins,[a] Richard M. Fogarty,[b] Lennart Frankemoelle,[c] Rebecca Rowe,[c] Coby J. Clarke,[d] David A. Duncan,[e] Robert G. Palgrave,[f] Roger A. Bennett,[a] Patricia A. Hunt,[g] and Kevin R. J. Lovelock.*[a]

[a] Department of Chemistry, University of Reading, Reading, RG6 6AD, UK
[b] Department of Materials, Imperial College London, SW7 2AZ, UK
[c] Department of Chemistry, Imperial College London, SW7 2AZ, UK
[d] School of Chemistry, University of Nottingham, Nottingham, NG7 2RD, UK
[e] Diamond Light Source, Didcot, Oxfordshire, OX11 0DE, UK
[f] Department of Chemistry, University College London, UK
[g] School of Chemical and Physical Sciences, Victoria University of Wellington, NZ
* Contact E–mail: k.r.j.lovelock@reading.ac.uk

1. Ionic liquids studied and synthesis S2-S5
2. XPS Apparatus S6
3. Computational details S7-S9
4. Data analysis. Peak fitting core XP spectra S10-S11
5. Data analysis. Peak fitting valence XP spectra S12
6. Data analysis. Charge referencing procedures S13-S15
7. Data analysis. AO photoionization cross-sections S16
8. Data analysis. Area normalisation S17
9. Results. XPS: demonstrating purity S18-S27
10. Results. XPS: valence XPS for $[C_8C_1Im][SnCl_3]$ versus perovskites S28
11. Results. XPS: cation contributions to valence XPS S29
12. Results. DFT: atomic orbital contributions to molecular orbitals S30-S34
13. Results. XPS: variable $h\nu$ valence XPS data for $[C_8C_1Im][SnCl_3]$ S35-S38
14. Results. XPS versus Gelius-weighted DoS S39-S42
15. Results. DFT: tDoS, pDoS and MO representations S43-S45
16. Results. XPS: valence XPS for $[Sn(CF_3SO_3)_3]^-$ versus $[CF_3SO_3]^-$ S46
17. Results. XPS: core XPS for $[Sn(CF_3SO_3)_3]^-$ versus $[SnBr_3]^-$ and $[SnBr_3]^-$ S47
18. Results. XPS: E_B(cation core) vs. E_B(anion valence) comparisons S48
19. Results. DFT: atomic charges S49
20. Results. DFT: coordinates S50
21. References S51-S52

1. Ionic Liquids Studied and Synthesis

$[C_8C_1Im]_2[Sn(CF_3SO_3)_3][CF_3SO_3]$ was prepared using the following method. 1-Octyl-3-methylimidazolium trifluoromethylsulfonate, $[C_8C_1Im][CF_3SO_3]$, was purchased from Iolitec (99%), and was dried on a Schlenk line at $<2 \times 10^{-2}$ mbar at 70°C for 48 h before being transferred to a LABstar (MBraun) glovebox with <0.5 ppm of O_2 and H_2O. $[C_8C_1Im][CF_3SO_3]$ was gently heated to 40°C; next, 2-3 g of $[C_8C_1Im][CF_3SO_3]$ was decanted into a 30 mL screw top glass vial. The desired amount of tin(II) trifluoromethylsulfonate, $Sn(CF_3SO_3)_2$, (Thermo Scientific, 98%, used as received) was calculated and weighed into a vial, a stirrer bar was added, and the temperature was raised to 70°C. The vial was periodically handled to wash any solid particles from the sides until a clear solution persisted (typically < 48 h). For both laboratory and synchrotron measurements, the samples were mounted in air; exposure to air was limited to <10 minutes.

Synthesis for $[C_8C_1Im][CF_3SO_3]$ was reported in reference [1]. Syntheses for 14 of the 16 ILs studied here were reported in reference [2].

Table S1. ILs investigated in this work.

IL no.	Abbreviation	Structure	Name
1	$[C_8C_1Im]Cl$	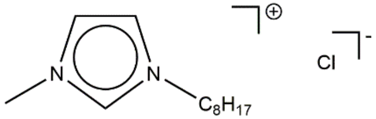	1–octyl–3–methylimidazolium chloride
2	$[C_8C_1Im]Br$	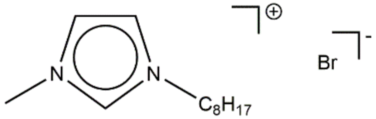	1–octyl–3–methylimidazolium bromide
3	$[C_8C_1Im][SnCl_3]$	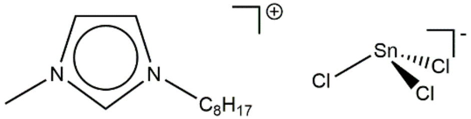	1-octyl-3-methylimidazolium trichlorostannate
4	$[C_8C_1Im][SnBr_3]$	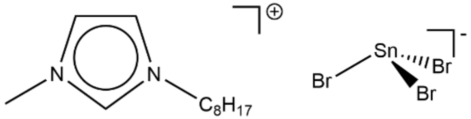	1-octyl-3-methylimidazolium tribromostannate
5	$[C_8C_1Im]_2[Sn(CF_3SO_3)_3][CF_3SO_3]$	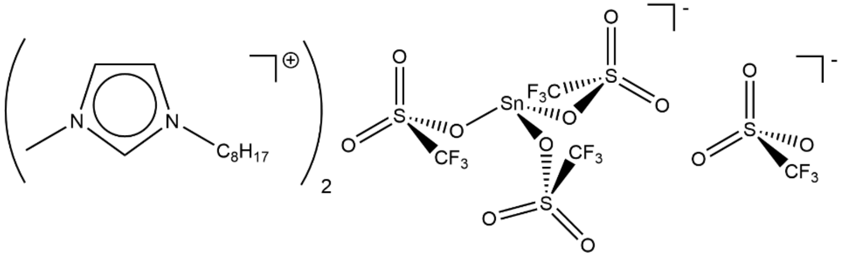	1-octyl-3-methylimidazolium tri(trifluoromethylsulfonate)stannate + 1-octyl-3-methylimidazolium trifluoromethylsulfonate
6	$[C_8C_1Im]_2[Bi_2Cl_8]$	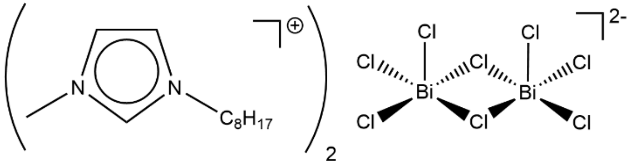	bis(1-octyl-3-methylimidazolium) octachlorodibismuthate
7	$[C_8C_1Im]_2[ZnCl_4]$	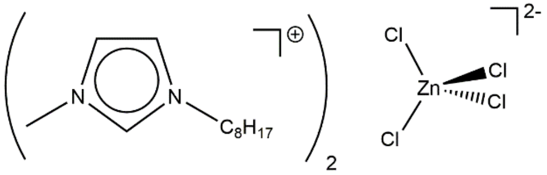	bis(1-octyl-3-methylimidazolium) tetrachlorozincate

8	$[C_8C_1Im]_2[ZnBr_4]$	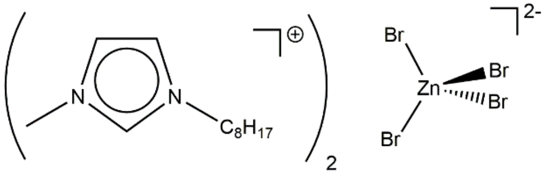	bis(1-octyl-3-methylimidazolium) tetrabromozincate
9	$[C_8C_1Im]_2[Zn_2Br_6]$	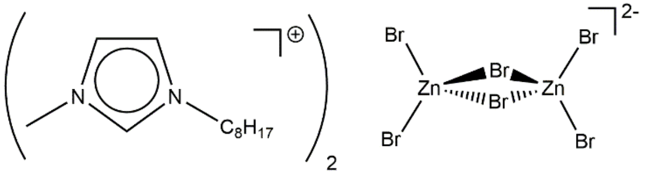	bis(1-octyl-3-methylimidazolium) hexabromodizincate
10	$[C_8C_1Im]_2[Zn_2Cl_6]$	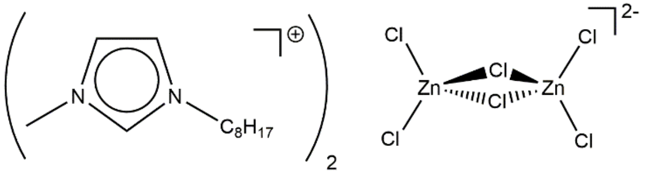	bis(1-octyl-3-methylimidazolium) hexachlorodizincate
11	$[C_8C_1Im]_2[Zn_3Br_8]$	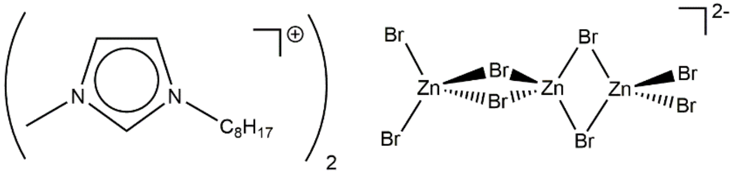	bis(1-octyl-3-methylimidazolium) octabromotrizincate
12	$[C_8C_1Im]_2[Zn_4Br_{10}]$	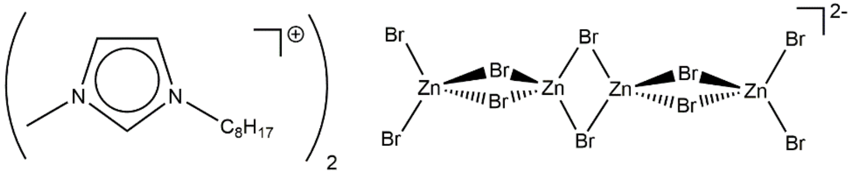	bis(1-octyl-3-methylimidazolium) decabromotetrazincate
13	$[C_8C_1Im]_2[Zn_4Cl_{10}]$	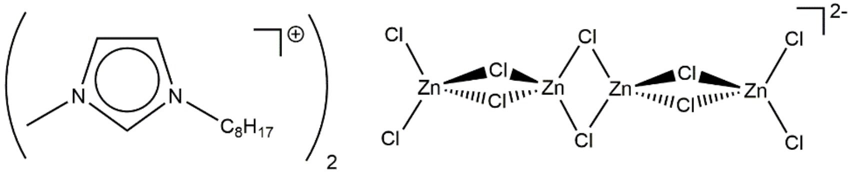	bis(1-octyl-3-methylimidazolium) decachlorotetrazincate [b]

14	$[C_8C_1Im][InCl_4]$	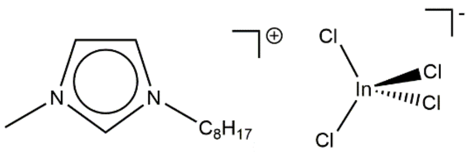	1-octyl-3-methylimidazolium tetrachloroindate
15	$[C_8C_1Im][InBr_4]$	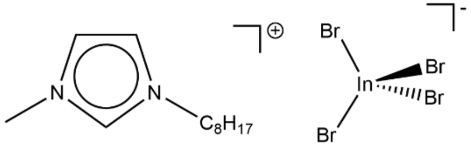	1-octyl-3-methylimidazolium tetrabromoindate
16	$[C_8C_1Im][CF_3SO_3]$	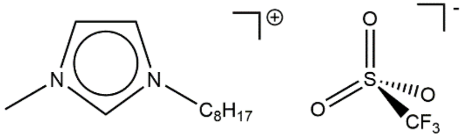	1-octyl-3-methylimidazolium trifluoromethylsulfonate

2. XPS Apparatus

Laboratory XPS

Laboratory XP spectra for 15 ILs are used in this paper. Core and valence XP spectra were published for 11 ILs in the ESI of reference [3]. Core and valence XP spectra are published for the first time for $[C_8C_1Im][SnBr_3]$, $[C_8C_1Im]_2[Sn(CF_3SO_3)_3][CF_3SO_3]$, $[C_8C_1Im]_2[Bi_2Cl_8]$ and $[C_8C_1Im][InBr_4]$. Laboratory-based XPS was recorded for these four ILs $[C_8C_1Im][SnBr_3]$, $[C_8C_1Im]_2[Sn(CF_3SO_3)_3][CF_3SO_3]$, $[C_8C_1Im]_2[Bi_2Cl_8]$ and $[C_8C_1Im][InBr_4]$) at the University of Reading on a Thermo Scientific ESCALAB 250 monochromated Al Kα source ($h\nu$ = 1486.6 eV) spectrometer. A drop of IL was placed directly onto a stainless steel sample plate. This sample was placed in a loadlock and the pressure reduced to 10^{-7} mbar by pumping down for > 6 hours. After attaining the required pressure, the IL was transferred to the analysis chamber. Etching was carried out using a rastered 500 eV Ar^+ ion beam (20 seconds for $[C_8C_1Im]_2[Bi_2Cl_8]$). Acquisition parameters were matched to give comparable energy resolution with data already published; a pass energy of 20 eV was used for core-levels.

Synchrotron XPS

The soft and hard synchrotron XPS was performed on the I09 beamline at Diamond Light Source (UK).[4] A thin film (less than 0.1 ml, essentially so no drop could be observed) of the IL sample was placed on a tantalum sample holder and attached to a standard Omicron sample holder. The XP spectra were acquired using a VG Scienta EW4000 HAXPES analyser, which had an angular acceptance of ±30°. The analyser was mounted with its centre approximately 80° (incident soft X-ray light) and 85° (incident hard X-ray light) away from the direction of the incident X-ray light (with respect to the sample) in the plane of the photon polarisation (linear horizontal); the analyser slits (and thus the angular acceptance direction) were also in the plane of the photon polarisation. Due to significant observable beam damage, the flux of the synchrotron light was decreased by first defocussing the incident light (~20 fold for soft X-rays and ~100 fold for hard X-rays) and by detuning the undulator (*i.e*. offsetting the undulator gap) away from the maximum intensity so as to detune the flux a further 100-fold. Prior to XPS measurements the sample was Ar^+ sputtered for 30 minutes at a voltage of 500 V.

3. Computational details

3.1. General details

Lone ion DFT calculations were carried out using Becke's three-parameter exchange functional in combination with the Lee, Yang and Parr correlation functional (B3LYP) as implemented in the Gaussian 16 or 09 suite of programs.[5-8] Grimme's D3 dispersion correction with Becke-Johnson damping was used to account for dispersion.[9-12] The combination of B3LYP with D3-BJ damping is referred to as the B3LYP-D3(BJ) functional henceforth. LANL2DZ pseudo potentials and the associated basis sets were employed for Zn, Sn and In. The 6-311+G(d,p) basis set was employed for Cl in these calculations. Data for $[Bi_2Cl_8]^{2-}$ anion was taken from calculations reported in reference [13]; cc-pVDZ-pp (scalar relativistic) pseudopotentials and aug-cc-pVDZ associated basis sets were employed for the heavy Bi atoms.[14, 15] The aug-cc-pVDZ basis set was employed for Cl in these calculations. Optimisations were carried out under no symmetry constraints. The SCF convergence criteria were 10^{-9} on the density matrix and 10^{-7} on the energy matrix. The numerical integration grid was a pruned grid with 99 radial shells and 590 angular points per shell. Vibrational frequencies were evaluated to confirm structures are minima. Two difficult convergence cases are discussed below. A polarisable continuum medium parameterised for ILs (SMD[16]) was used. The SMD parameters employed are shown in Table S2; IL SMD parameters were used for all IL ion calculations.[13, 16, 17]

$[ZnCl_4]^{2-}$ and $[SnCl_3]^-$ in the gas phase converge well; however, with the SMD environment neither structure fully converges, but does reach a loose convergence. The gas-phase structure was taken and optimised with the SMD environment. In the case of $[ZnCl_4]^{2-}$ the structure with the lowest force prior to dissociation was selected and a frequency analysis carried out. Forces are converged to a root mean square (RMS) of 0.0004, the lowest formally zero frequency is -52 cm^{-1} while the lowest frequency is 45 cm^{-1}. Thus, the structure is loosely converged. In the case of $[SnCl_3]^-$ the structure remained bound, and energies oscillated within 2 kJ mol^{-1}. This finding is not uncommon when using SMD with an empirical dispersion correction. The step with the lowest RMS force of 0.0002 (and within the cut-off criteria) was selected for frequency analysis, the lowest formally zero frequency is -40 cm^{-1} while the lowest frequency is 92 cm^{-1}.

3.2. SMD parameters

Table S2. Parameters used in the SMD model (apart from for $[Bi_2Cl_8]^{2-}$ where slightly different SMD parameters were used[13, 17])

Solvent	Relative permittivity, ε	Refractive index, n	Surface tension, γ (cal mol^{-1} $Å^2$)	Abraham acidity, α	Abraham basicity, β
$[C_4C_1Im][PF_6]$	11.40	1.4090	70.24	0.266	0.216

3.3. DoS, pDoS and Gelius-weighted DoS

All density of states (DoS), partial DoS (pDoS) and Gelius-weighted DoS were generated for the lowest energy conformer for each system. The DoS of a system is defined as:

3.3.1. DoS

$$DoS(E_B) = \sum_i F(E_B - (-\xi_i))$$

where F is a broadening function and E_B is binding energy (the negative of energy, positive binding energies indicate stable states). A GL30 (70% Gaussian, 30% Lorentzian) broadening function (with full width at half maximum, FWHM, dependent on the data set) was used for all DoS plots.

3.3.2. Gaussian-Lorentzian broadening

The exact form of the broadening function (referred to as GL30) used in DoS, pDoS and Gelius-weighted DoS is shown below (along with the formula for the DoS).

$$DoS(E_B) = \sum_i F(E_B - (-\xi_i))$$

$$F(E_B - (-\xi_i)) = 0.7G(E_B - (-\xi_i)) + 0.3L(E_B - (-\xi_i))$$

$$G(E_B - (-\xi_i)) = \frac{1}{\eta\sqrt{2\pi}} exp(\frac{-(E_B - (-\xi_i))^2}{2\eta^2})$$

$$L(E_B - (-\xi_i)) = \frac{1}{\pi}\frac{0.5\Gamma}{(E_B - (-\xi_i))^2 + 0.5\Gamma^2}$$

$$\Gamma = FWHM$$

$$\eta = \frac{\Gamma}{2\sqrt{2ln2}}$$

A broadening function was used for all calculated spectra. For calculations compared to laboratory XP spectra ($h\nu$ = 1486.6 eV) for $[C_8C_1Im][SnCl_3]$, $[C_8C_1Im]_2[ZnCl_4]$, $[C_8C_1Im][InCl_4]$ and $[C_8C_1Im]Cl$, FWHM = 1.0 eV. For calculations compared to laboratory XP spectra ($h\nu$ = 1486.6 eV) for halobismuthate-based ILs, FWHM = 1.3 eV. The larger FWHM used for the halobismuthate anions reflects the greater range of speciation for the halobismuthate anions compared to the other anions studied here, as explained in Section 11. For calculations compared to synchrotron XP spectra for $[C_8C_1Im][SnCl_3]$, FWHM = 0.8 eV for $h\nu$ = 250 eV and FWHM = 1.2 eV for $h\nu$ = 6000 eV. These values were based on the experimental FWHM for $[C_8C_1Im][SnCl_3]$; FWHM(Cl 3p + Sn 5s + Sn 5p anti-bonding) and FWHM(Sn $4d_{5/2}$) were used (ESI Table S14).

3.3.3. pDoS

The pDoS of an atomic orbital *k* is defined as:

$$pDoS(k, E_B) = \sum_i n_{k,i} F(E_B - (-\xi_i))$$

where $n_{k,i}$ is the fractional contribution of AO *k* to MO *i*. The value of $n_{k,i}$ was determined using Mulliken population analysis as implemented in multiwfn.[18,19] The pDOS for a fragment (*e.g.* the anion) was obtained by summing the pDoS for all AOs centred on that fragment.

3.3.4. Gelius-weighted DoS

The Gelius approximation allows the photoionization cross-section of each MO to be written as a linear combination of the contributing AOs multiplied by their atomic photoionization cross-sections.[20, 21] Therefore, the intensity, *I*, of a calculated spectrum can be written:

$$I(E_B) = \sum_k \sum_i \sigma_k n_{k,i} F(E_B - (-\xi_i)) = \sum_k \sigma_k . pDoS(k, E_B)$$

where σ_k is the photoionization cross–section of AO *k*.

Both Yeh and Lindau and Scofield atomic photoionization cross-sections were used in this paper.[22, 23] For both laboratory-based XPS apparatus used here, the source-detector angle was ~54.7°, *i.e*. the magic angle. At this angle, the anisotropy parameter was zero.[24, 25] Therefore, the asymmetry parameter component was not considered when analysing the laboratory-based, $h\nu$=1486.6 eV XPS results. For the synchrotron-based XPS apparatus used here, the source-detector angle was ~87° (incident soft X-ray light) and ~93° (incident hard X-ray light), *i.e*. significantly away from the magic angle. At these angles, the anisotropy parameter was non-zero.[24, 25] Therefore, the asymmetry parameter component was considered when analysing the synchrotron-based XPS results by using Equation 2 given in reference [26]; anisotropy parameters for soft XPS were taken from reference [22] and anisotropy parameters for hard XPS were extrapolated from those in reference [22]. Equation 2 given in reference [26] was developed for the hard XPS apparatus on the I09 end-station. However, the soft XPS apparatus had a very similar source-detector angle (only ~5° difference); hence, Equation 2 given in reference [26] is used for Gelius-weighted DoS calculations for comparisons to both soft and hard XPS.

3.3.5. E_B shifts of DoS to match XPS experiments

A constant linear shift in E_B was applied to calculated DoS to align the valence states composed of large Cl 3p contributions in the experimental XP spectra and the calculated DoS (Figure 1). The E_B shifts were -2.88 eV for $[SnCl_3]^-$, $[InCl_4]^-$, $[ZnCl_4]^{2-}$ and -2.58 eV for the halobismuthate anions.

4. Data analysis. Peak fitting core level XP spectra

All core XP spectra were fitted using CASAXPS™ software. Spectra were fitted with a GL30 lineshape (70% Gaussian, 30% Lorentzian) and a Shirley background. The constraints used to fit the core XP spectra are given in Table S3.

Peak fitting core level XP spectra is important for demonstrating purity (ESI Section 9). For this work peak fitting core level XP spectra is equally important for charge referencing.

Table S3. Fitting constraints used for core level X-ray photoelectron spectroscopy (XPS) for each ionic liquid

IL no.	IL	Core level	Fitting constraints used
1	$[C_8C_1Im]Cl$	C 1s	Peak area ratio 1:4:7 for C^2:C_{hetero}:C_{alkyl}
		Cl 2p	Peak area ratio 1:2 for $2p_{1/2}$:$2p_{3/2}$
			ΔE_B(Cl $2p_{3/2}$ - Cl $2p_{3/2}$) = 1.60 eV
2	$[C_8C_1Im]Br$	C 1s	Peak area ratio 1:4:7 for C^2:C_{hetero}:C_{alkyl}
		Br 3d	Peak area ratio 2:3 for $3d_{3/2}$:$3d_{5/2}$
			ΔE_B(Br $3d_{5/2}$ - Br $3d_{3/2}$) = 1.04 eV
3	$[C_8C_1Im][SnCl_3]$	C 1s	Peak area ratio 1:4:7 for C^2:C_{hetero}:C_{alkyl}
		Cl 2p	Peak area ratio 1:2 for $2p_{1/2}$:$2p_{3/2}$
			ΔE_B(Cl $2p_{3/2}$ - Cl $2p_{3/2}$) = 1.60 eV
		Sn 4d	Area constraint. 2:3 for Sn $4d_{3/2}$:Sn $4d_{5/2}$
4	$[C_8C_1Im][SnBr_3]$	C 1s	Peak area ratio 1:4:7 for C^2:C_{hetero}:C_{alkyl}
		Br 3d	Peak area ratio 2:3 for $3d_{3/2}$:$3d_{5/2}$
			ΔE_B(Br $3d_{5/2}$ - Br $3d_{3/2}$) = 1.04 eV
		Sn 4d	Area constraint. 2:3 for Sn $4d_{3/2}$:Sn $4d_{5/2}$
5	$[C_8C_1Im]_2[Sn(CF_3SO_3)_3][CF_3SO_3]$	C 1s	Peak area ratio 1:4:7 for C^2:C_{hetero}:C_{alkyl}
		S 2p	Peak area ratio 1:2 for $2p_{1/2}$:$2p_{3/2}$
		Sn 4d	Area constraint. 2:3 for Sn $4d_{3/2}$:Sn $4d_{5/2}$
6	$[C_8C_1Im][BiCl_4]$	C 1s	Peak area ratio 1:4:7 for C^2:C_{hetero}:C_{alkyl}
		Cl 2p	Peak area ratio 1:2 for $2p_{1/2}$:$2p_{3/2}$
7	$[C_8C_1Im]_2[ZnCl_4]$	C 1s	Peak area ratio 1:4:7 for C^2:C_{hetero}:C_{alkyl}
		Cl 2p	Peak area ratio 1:2 for $2p_{1/2}$:$2p_{3/2}$
			ΔE_B(Cl $2p_{3/2}$ - Cl $2p_{3/2}$) = 1.60 eV
10	$[C_8C_1Im]_2[Zn_2Cl_6]$	C 1s	Peak area ratio 1:4:7 for C^2:C_{hetero}:C_{alkyl}
		Cl 2p	Peak area ratio 1:2 for $2p_{1/2}$:$2p_{3/2}$
			Peak area ratio 1:2 for $Cl_{bridging}$:$Cl_{terminal}$
			ΔE_B(Cl $2p_{3/2}$ - Cl $2p_{3/2}$) = 1.60 eV
			FWHM($Cl_{terminal}$ $2p_{3/2}$) = FWHM($Cl_{terminal}$ $2p_{1/2}$)
			FWHM($Cl_{bridging}$ $2p_{3/2}$) = FWHM($Cl_{bridging}$ $2p_{1/2}$)
13	$[C_8C_1Im]_2[Zn_4Cl_{10}]$	C 1s	Peak area ratio 1:4:7 for C^2:C_{hetero}:C_{alkyl}
		Cl 2p	Peak area ratio 1:2 for $2p_{1/2}$:$2p_{3/2}$
			Peak area ratio 3:2 for $Cl_{bridging}$:$Cl_{terminal}$
			ΔE_B(Cl $2p_{3/2}$ - Cl $2p_{3/2}$) = 1.60 eV
			FWHM($Cl_{terminal}$ $2p_{3/2}$) = FWHM($Cl_{terminal}$ $2p_{1/2}$)
			FWHM($Cl_{bridging}$ $2p_{3/2}$) = FWHM($Cl_{bridging}$ $2p_{1/2}$)
8	$[C_8C_1Im]_2[ZnBr_4]$	C 1s	Peak area ratio 1:4:7 for C^2:C_{hetero}:C_{alkyl}
		Br 3d	Peak area ratio 2:3 for $3d_{3/2}$:$3d_{5/2}$
			ΔE_B(Br $3d_{5/2}$ - Br $3d_{3/2}$) = 1.04 eV
9	$[C_8C_1Im]_2[Zn_2Br_6]$	C 1s	Peak area ratio 1:4:7 for C^2:C_{hetero}:C_{alkyl}
		Br 3d	Peak area ratio 2:3 for $3d_{3/2}$:$3d_{5/2}$
			Peak area ratio 1:2 for $Br_{bridging}$:$Br_{terminal}$
			ΔE_B(Br $3d_{5/2}$ - Br $3d_{3/2}$) = 1.04 eV
			FWHM($Br_{terminal}$ $3d_{5/2}$) = FWHM($Br_{terminal}$ $3d_{3/2}$)
			FWHM($Br_{bridging}$ $3d_{5/2}$) = FWHM($Br_{bridging}$ $3d_{3/2}$)
11	$[C_8C_1Im]_2[Zn_3Br_8]$	C 1s	Peak area ratio 1:4:7 for C^2:C_{hetero}:C_{alkyl}
		Br 3d	Peak area ratio 2:3 for $3d_{3/2}$:$3d_{5/2}$
			Peak area ratio 1:1 for $Br_{bridging}$:$Br_{terminal}$
			ΔE_B(Br $3d_{5/2}$ - Br $3d_{3/2}$) = 1.04 eV
			FWHM($Br_{terminal}$ $3d_{5/2}$) = FWHM($Br_{terminal}$ $3d_{3/2}$)
			FWHM($Br_{bridging}$ $3d_{5/2}$) = FWHM($Br_{bridging}$ $3d_{3/2}$)

12	$[C_8C_1Im]_2[Zn_4Br_{10}]$	C 1s	Peak area ratio 1:4:7 for C^2:C_{hetero}:C_{alkyl}
		Br 3d	Peak area ratio 2:3 for $3d_{3/2}$:$3d_{5/2}$<br>Peak area ratio 3:2 for $Br_{bridging}$:$Br_{terminal}$<br>ΔE_B(Br $3d_{5/2}$ - Br $3d_{3/2}$) = 1.04 eV<br>FWHM($Br_{terminal}$ $3d_{5/2}$) = FWHM($Br_{terminal}$ $3d_{3/2}$)<br>FWHM($Br_{bridging}$ $3d_{5/2}$) = FWHM($Br_{bridging}$ $3d_{3/2}$)
14	$[C_8C_1Im][InCl_4]$	C 1s	Peak area ratio 1:4:7 for C^2:C_{hetero}:C_{alkyl}
		Cl 2p	Peak area ratio 1:2 for $2p_{1/2}$:$2p_{3/2}$<br>ΔE_B(Cl $2p_{3/2}$ - Cl $2p_{3/2}$) = 1.60 eV
15	$[C_8C_1Im][InBr_4]$	C 1s	Peak area ratio 1:4:7 for C^2:C_{hetero}:C_{alkyl}
		Br 3d	Peak area ratio 2:3 for $3d_{3/2}$:$3d_{5/2}$<br>ΔE_B(Br $3d_{5/2}$ - Br $3d_{3/2}$) = 1.04 eV
16	$[C_8C_1Im][CF_3SO_3]$	C 1s	Peak area ratio 1:4:7 for C^2:C_{hetero}:C_{alkyl}
		S 2p	Peak area ratio 1:2 for $2p_{1/2}$:$2p_{3/2}$

5. Data analysis. Peak fitting valence XP spectra

5.1. Choosing the number of components to fit for valence XP spectra

For valence XP spectra, the differences in the FWHM were due to the contributions from different MOs. The source/detector contributions were essentially constant for the laboratory XPS experiments performed on the same XPS apparatus, and the contribution from lifetime broadening were relatively small for valence XPS. Sn 4d, In 4d and Bi 5d AOs are usually considered core states; Zn 3d AOs can be classified as either core or valence states.[27] Therefore, these metal states were used as a guide to the FWHM of a single valence state. Moreover, the MOs from the DFT calculations were also used to aid peak fitting and identify different contributions to the valence electronic structure.

5.2. Peak fitting constraints used for valence XP spectra

All valence XP spectra were fitted using CASAXPS™ software. Spectra were fitted with a GL30 lineshape (70% Gaussian, 30% Lorentzian) and a Shirley background. The constraints used to fit the valence XP spectra are given in Table S4.

Table S4. Peak fitting constraints used for valence level X-ray photoelectron spectroscopy (XPS) for each ionic liquid

IL no.	IL	$h\nu$ / eV	Peak fitting constraints used
1	$[C_8C_1Im]Cl$	1486.6	No constraints
2	$[C_8C_1Im]Cl$	1486.6	No constraints
3	$[C_8C_1Im][SnCl_3]$	1486.6	FWHM(Cl 3p + Sn 5p bonding) = FWHM(Cl 3p weakly bonding/non-bonding) = FWHM(Cl 3p weakly anti-bonding)
3	$[C_8C_1Im][SnCl_3]$	250	FWHM(Cl 3p + Sn 5p bonding) = FWHM(Cl 3p weakly bonding/non-bonding/weakly anti-bonding)
3	$[C_8C_1Im][SnCl_3]$	6000	FWHM(Cl 3p + Sn 5p bonding) = FWHM(Cl 3p weakly bonding/non-bonding/weakly anti-bonding)
4	$[C_8C_1Im][SnBr_3]$	1486.6	FWHM(Br 4p + Sn 5p bonding) = FWHM(Sn 5p weakly bonding/non-bonding) = FWHM(Br 4p weakly anti-bonding)
6	$[C_8C_1Im]_2[Bi_2Cl_8]$	1486.6	FWHM(Cl 3p + Bi 6p bonding) = FWHM(Cl 3p weakly bonding/non-bonding) = FWHM(Cl 3p weakly anti-bonding) = FWHM(Cl 3p + Bi 6s/6p bonding)
7	$[C_8C_1Im]_2[ZnCl_4]$	1486.6	FWHM(Cl 3p + Zn 4p bonding) = FWHM(Cl 3p weakly bonding/non-bonding) = FWHM(Cl 3p weakly anti-bonding)
8	$[C_8C_1Im]_2[ZnBr_4]$	1486.6	FWHM(Br 4p + Zn 4p bonding) = FWHM(Br 4p weakly bonding/non-bonding) = FWHM(Br 4p weakly anti-bonding)
9	$[C_8C_1Im]_2[Zn_2Cl_6]$	1486.6	FWHM(Cl 3p + Zn 4p bonding) = FWHM(Cl 3p weakly bonding/non-bonding) = FWHM(Cl 3p weakly anti-bonding)
10	$[C_8C_1Im]_2[Zn_2Br_6]$	1486.6	FWHM(Br 4p + Zn 4p bonding) = FWHM(Br 4p weakly bonding/non-bonding) = FWHM(Br 4p weakly anti-bonding)
11	$[C_8C_1Im]_2[Zn_3Br_8]$	1486.6	FWHM(Br 4p + Zn 4p bonding) = FWHM(Br 4p weakly bonding/non-bonding) = FWHM(Br 4p weakly anti-bonding)
12	$[C_8C_1Im]_2[Zn_4Cl_{10}]$	1486.6	FWHM(Cl 3p + Zn 4p bonding) = FWHM(Cl 3p weakly bonding/non-bonding) = FWHM(Cl 3p weakly anti-bonding)
13	$[C_8C_1Im]_2[Zn_4Br_{10}]$	1486.6	FWHM(Br 4p + Zn 4p bonding) = FWHM(Br 4p weakly bonding/non-bonding) = FWHM(Br 4p weakly anti-bonding)
14	$[C_8C_1Im][InCl_4]$	1486.6	FWHM(Cl 3p + In 5p bonding) = FWHM(Cl 3p weakly bonding/non-bonding) = FWHM(Cl 3p weakly anti-bonding)
15	$[C_8C_1Im][InBr_4]$	1486.6	FWHM(Br 4p + In 5p bonding) = FWHM(Br 4p weakly bonding/non-bonding) = FWHM(Br 4p weakly anti-bonding)

6. Data analysis. Charge referencing procedures

All lab- and synchrotron-based XP spectra were effectively charge referenced to E_B(C_{alkyl} 1s) = 285.00 eV (Table S5). All XP spectra measured at $h\nu$ = 1486.6 eV were charge referenced directly to E_B(C_{alkyl} 1s) = 285.00 eV (Table S5). Once the valence XP spectrum for [C_8C_1Im][$SnCl_3$] measured at $h\nu$ = 1486.6 eV was charge referenced to E_B(C_{alkyl} 1s) = 285.00 eV, E_B(Sn $4d_{5/2}$) = 26.09 eV (ESI Figure S1). Therefore, E_B(Sn $4d_{5/2}$) = 26.09 eV was used to charge reference the valence XP spectra recorded at $h\nu$ = 250 eV and $h\nu$ = 6000 eV; this method effectively charge referenced the valence XP spectra to E_B(C_{alkyl} 1s) = 285.00 eV.

For lab XPS for the four ILs studied here ([C_8C_1Im][$SnBr_3$], [C_8C_1Im]$_2$[Sn(CF_3SO_3)$_3$][CF_3SO_3], [C_8C_1Im]$_2$[Bi_2Cl_8] and [C_8C_1Im][$InBr_4$]) an interpolation method, which has proven to be effective for charge referencing XP spectra for ILs,[1] was used to charge reference the data recorded at $h\nu$ = 1486.6 eV.

Table S5. Experimental X-ray photoelectron spectroscopy (XPS) details on the charge correction applied for each ionic liquid, and any fitting constraints needed to fit the core orbital used for charge referencing

IL no.	Abbreviation	$h\nu$ at which valence XPS measured at / eV	Core orbital used for charge referencing	Area fitting constraints used	E_B for core orbital used for charge referencing / eV	Rationale for choosing core orbital used for charge referencing
1	$[C_8C_1Im]Cl$	1486.6	C_{alkyl} 1s	1:4:7 for C^2:C_{hetero}:C_{alkyl}	285.0	Long alkyl chain
2	$[C_8C_1Im]Br$	1486.6	C_{alkyl} 1s	1:4:7 for C^2:C_{hetero}:C_{alkyl}	285.0	Long alkyl chain
3	$[C_8C_1Im][SnCl_3]$	1486.6	C_{alkyl} 1s	1:4:7 for C^2:C_{hetero}:C_{alkyl}	285.0	Long alkyl chain
3	$[C_8C_1Im][SnCl_3]$	250	Sn $4d_{5/2}$	2:3 for $4d_{3/2}$:$4d_{5/2}$	26.09	Core level at similar E_B to valence levels
3	$[C_8C_1Im][SnCl_3]$	6000	Sn $4d_{5/2}$	2:3 for $4d_{3/2}$:$4d_{5/2}$	26.09	Core level at similar E_B to valence levels
4	$[C_8C_1Im][SnBr_3]$	1486.6	C_{alkyl} 1s	1:4:7 for C^2:C_{hetero}:C_{alkyl}	285.0	Long alkyl chain
5	$[C_8C_1Im]_2[Sn(CF_3SO_3)_3][CF_3SO_3]$	1486.6	C_{alkyl} 1s	1:4:7 for C^2:C_{hetero}:C_{alkyl}	285.0	Long alkyl chain
6	$[C_8C_1Im]_2[Bi_2Cl_8]$	1486.6	C_{alkyl} 1s	1:4:7 for C^2:C_{hetero}:C_{alkyl}	285.0	Long alkyl chain
7	$[C_8C_1Im]_2[ZnCl_4]$	1486.6	C_{alkyl} 1s	1:4:7 for C^2:C_{hetero}:C_{alkyl}	285.0	Long alkyl chain
8	$[C_8C_1Im]_2[ZnBr_4]$	1486.6	C_{alkyl} 1s	1:4:7 for C^2:C_{hetero}:C_{alkyl}	285.0	Long alkyl chain
9	$[C_8C_1Im]_2[Zn_2Br_6]$	1486.6	C_{alkyl} 1s	1:4:7 for C^2:C_{hetero}:C_{alkyl}	285.0	Long alkyl chain
10	$[C_8C_1Im]_2[Zn_2Cl_6]$	1486.6	C_{alkyl} 1s	1:4:7 for C^2:C_{hetero}:C_{alkyl}	285.0	Long alkyl chain
11	$[C_8C_1Im]_2[Zn_3Br_8]$	1486.6	C_{alkyl} 1s	1:4:7 for C^2:C_{hetero}:C_{alkyl}	285.0	Long alkyl chain
12	$[C_8C_1Im]_2[Zn_4Br_{10}]$	1486.6	C_{alkyl} 1s	1:4:7 for C^2:C_{hetero}:C_{alkyl}	285.0	Long alkyl chain
13	$[C_8C_1Im]_2[Zn_4Cl_{10}]$	1486.6	C_{alkyl} 1s	1:4:7 for C^2:C_{hetero}:C_{alkyl}	285.0	Long alkyl chain
14	$[C_8C_1Im][InCl_4]$	1486.6	C_{alkyl} 1s	1:4:7 for C^2:C_{hetero}:C_{alkyl}	285.0	Long alkyl chain
15	$[C_8C_1Im][InBr_4]$	1486.6	C_{alkyl} 1s	1:4:7 for C^2:C_{hetero}:C_{alkyl}	285.0	Long alkyl chain

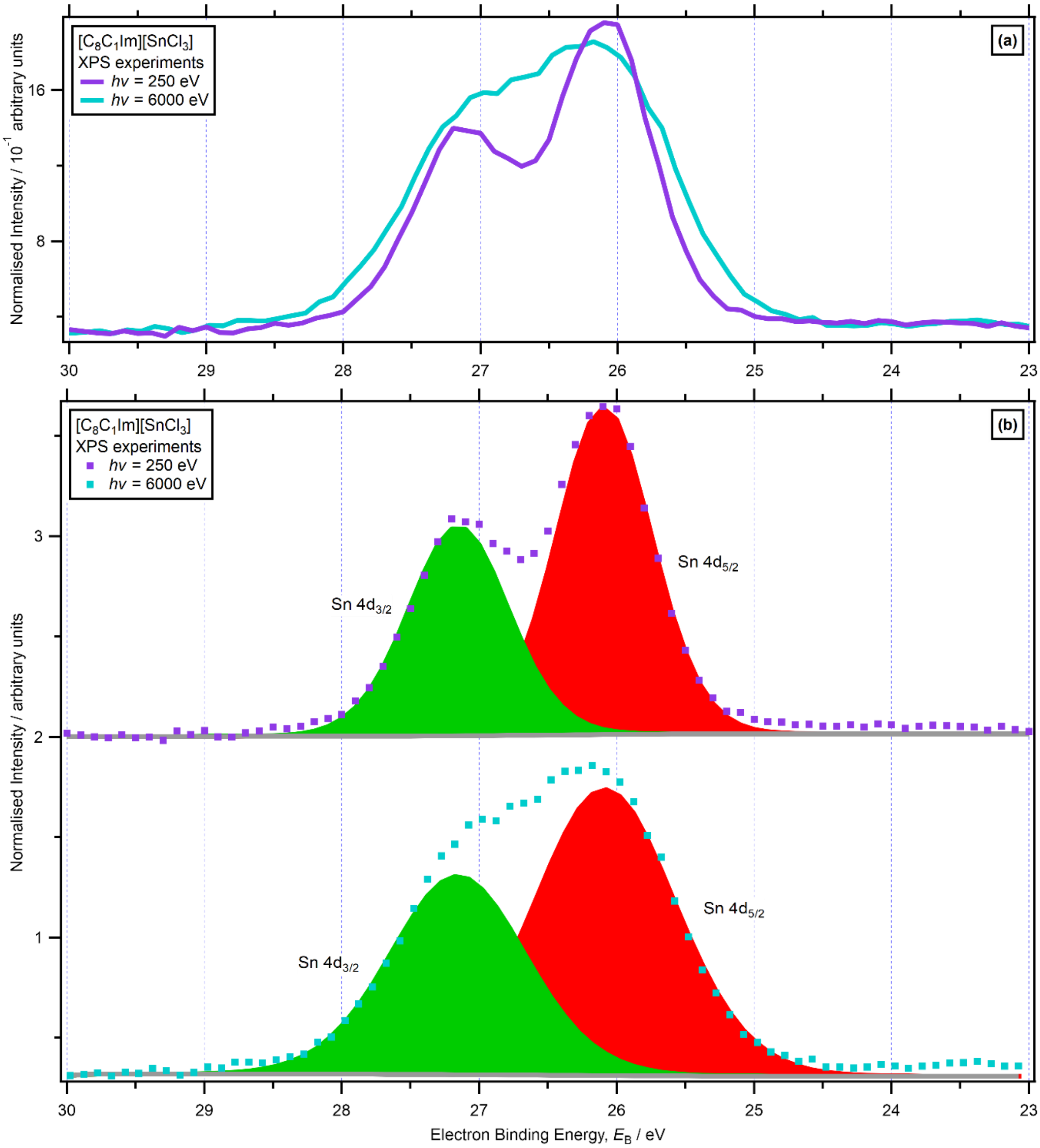

Figure S1. Area normalised synchrotron valence XP spectra for $[C_8C_1Im][SnCl_3]$ (recorded at $h\nu$ = 250 eV and 6000 eV): (a) overlaid, (b) vertically offset for clarity, with fitted components included. The data was charge referenced to E_B(Sn $4d_{5/2}$) = 26.09 eV.

7. Data analysis. AO photoionization cross-sections for valence XP spectra

Table S6. Calculated photoionization cross-sections at $h\nu$ = 1486.6 eV, taken from reference [22].

Atomic Orbital	Calculated photoionization cross-sections (at $h\nu$ = 1486.6 eV) / Barns
Sn 5s	1242
Sn 5p	774.7
Zn 3d	11170
Zn 4s	834.2
In 5s	1024
In 5p	273.7
Cl 3p	1952
Br 4p	4503
N 2p	90.72
C 2p	21.49
H 1s	2.755

Table S7. Calculated photoionization cross-sections at $h\nu$ = 250 eV and $h\nu$ = 6000 eV, taken from references [22, 23].

Atomic Orbital	Calculated photoionization cross-sections (at $h\nu$ = 250 eV) / Barn	Calculated photoionization cross-sections (at $h\nu$ = 6000 eV) / Barn	Times calculated photoionization cross-sections decreased from $h\nu$ = 250 eV to $h\nu$ = 6000 eV
Sn 5s	23980	88.190	272
Sn 5p	11520	36.454	316
Cl 3p	179800	19.959	9000
N 2p	36240	0.38027	95300
C 2p	10380	0.084094	123000

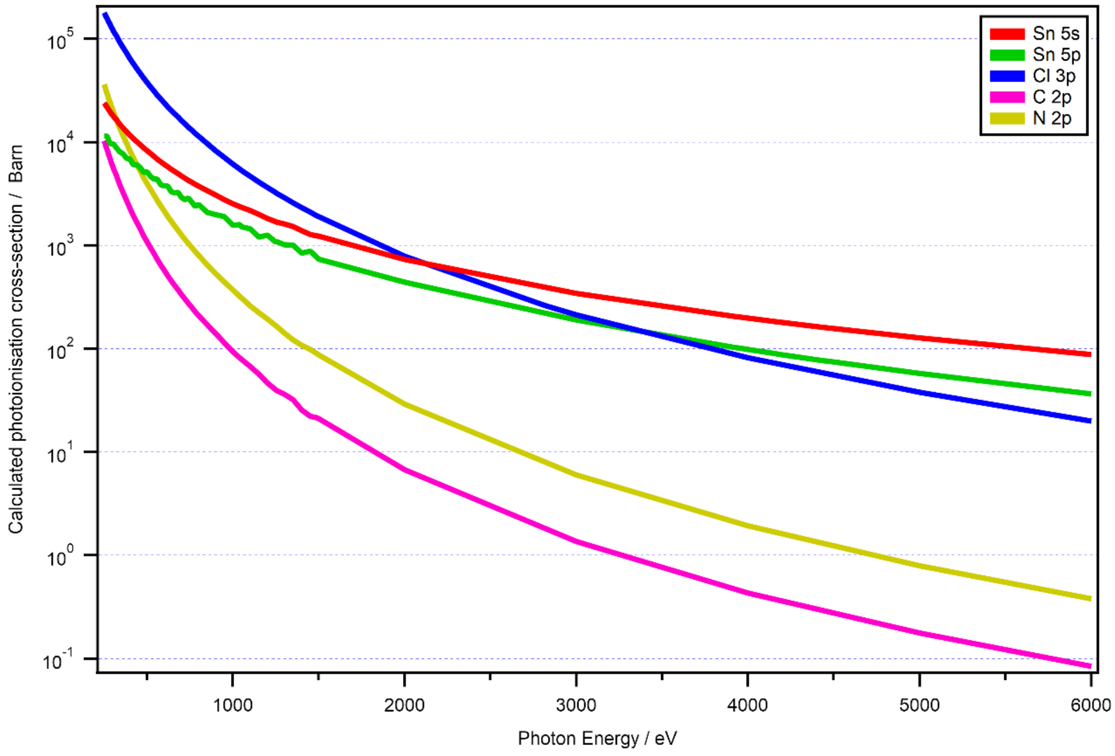

Figure S2. Calculated photoionization cross-sections from low $h\nu$ ($h\nu$ = 250 eV) to high $h\nu$ ($h\nu$ = 6000 eV), taken from references [22, 23]. Note the logarithmic y-scale.

8. Data analysis. Area normalisation

Valence XP spectra recorded at $h\nu$ = 250 eV and $h\nu$ = 6000 eV for [C_8C_1Im][$SnCl_3$] were area normalised by eye, to show the differences in relative component areas with respect to changing $h\nu$. Figure S3 shows the same data as Figure 1a, but with a wider E_B range.

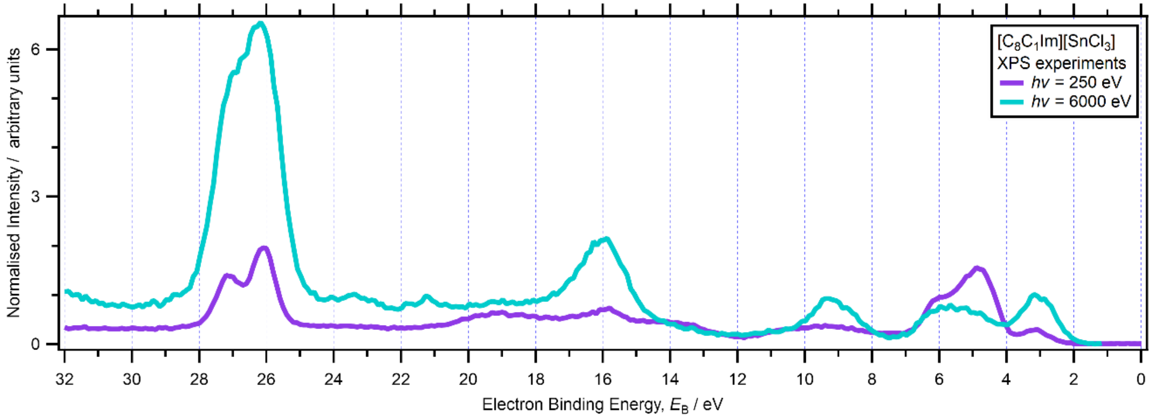

Figure S3. Area normalised experimental valence XP spectra for [C_8C_1Im][$SnCl_3$] at $h\nu$ = 250 eV and $h\nu$ = 6000 eV. All XP spectra were charge referenced to $E_B(C_{alkyl}$ 1s) = 285.00 eV. Further details on the procedures used for charge referencing of XP spectra are outlined in ESI Section 6.

9. Results. XPS: demonstrating purity

Ionic liquids have previously been shown, at times, to contain silicon-based contaminants.[28, 29] All of the ILs apart from one were free from contamination (Figure S4 to Figure S10); $[C_8C_1Im]_2[Bi_2Cl_8]$ showed a small Bi metal contamination, very likely from X-ray beam damage.

Given the relatively high flux of the synchrotron radiation, checks were made for changes over time. For the valence XP spectrum recorded at $h\nu$ = 6000 eV for $[C_8C_1Im][SnCl_3]$, Cl 1s recorded at $h\nu$ = 6000 eV before and after the valence XP spectrum showed no significant differences over time (Figure S11a). For the valence XP spectrum recorded at $h\nu$ = 250 eV for $[C_8C_1Im][SnCl_3]$, N 1s recorded at $h\nu$ = 870 eV before and after the valence XP spectrum showed no significant differences over time (Figure S11b).

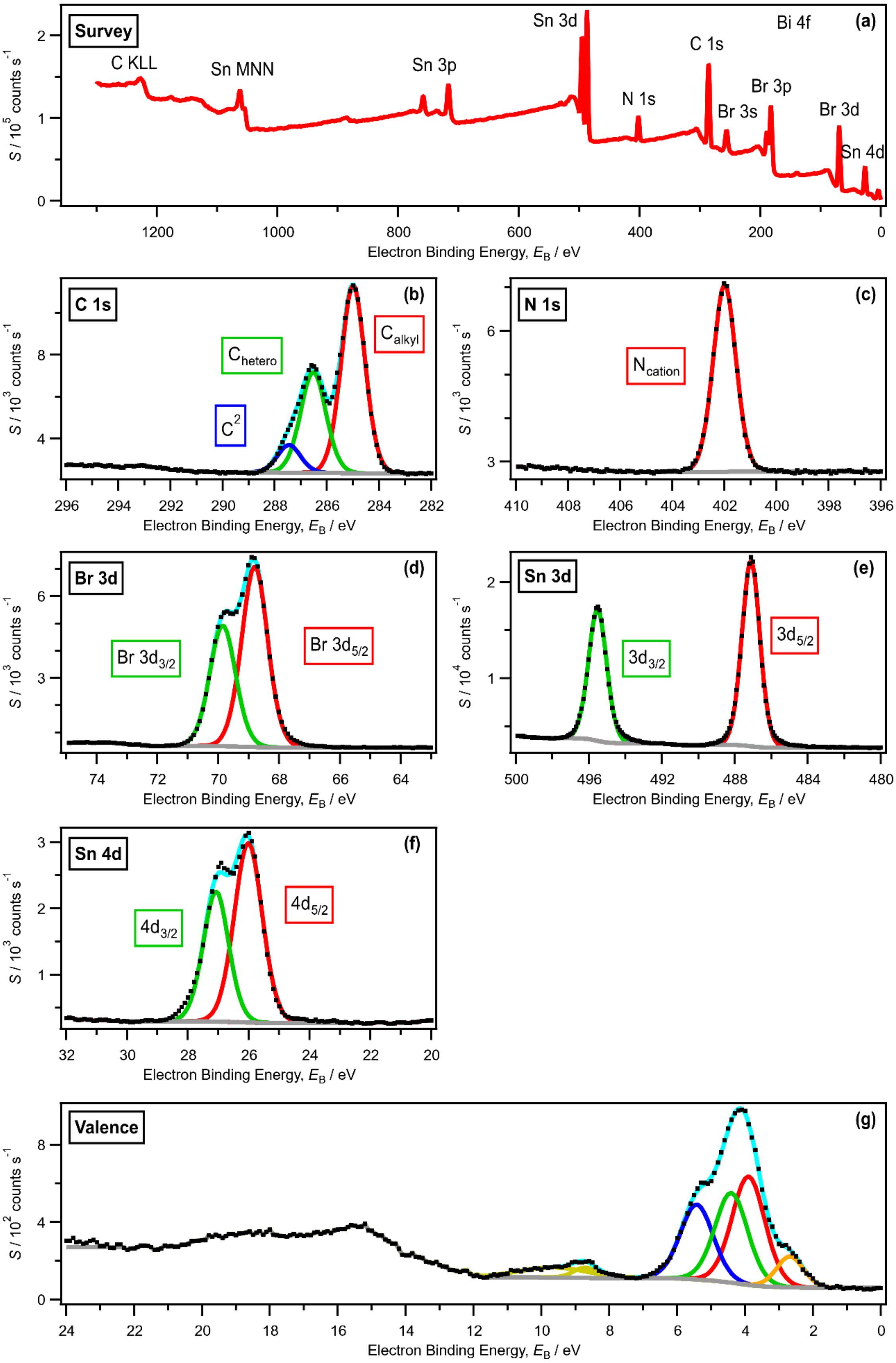

Figure S4. (a) Survey, (b-f) core and (g) valence XP spectra for $[C_8C_1Im][SnBr_3]$ recorded on laboratory–based XPS apparatus at $h\nu$ = 1486.6 eV. All electron spectra are charge referenced using procedures outlined in ESI Section 6.

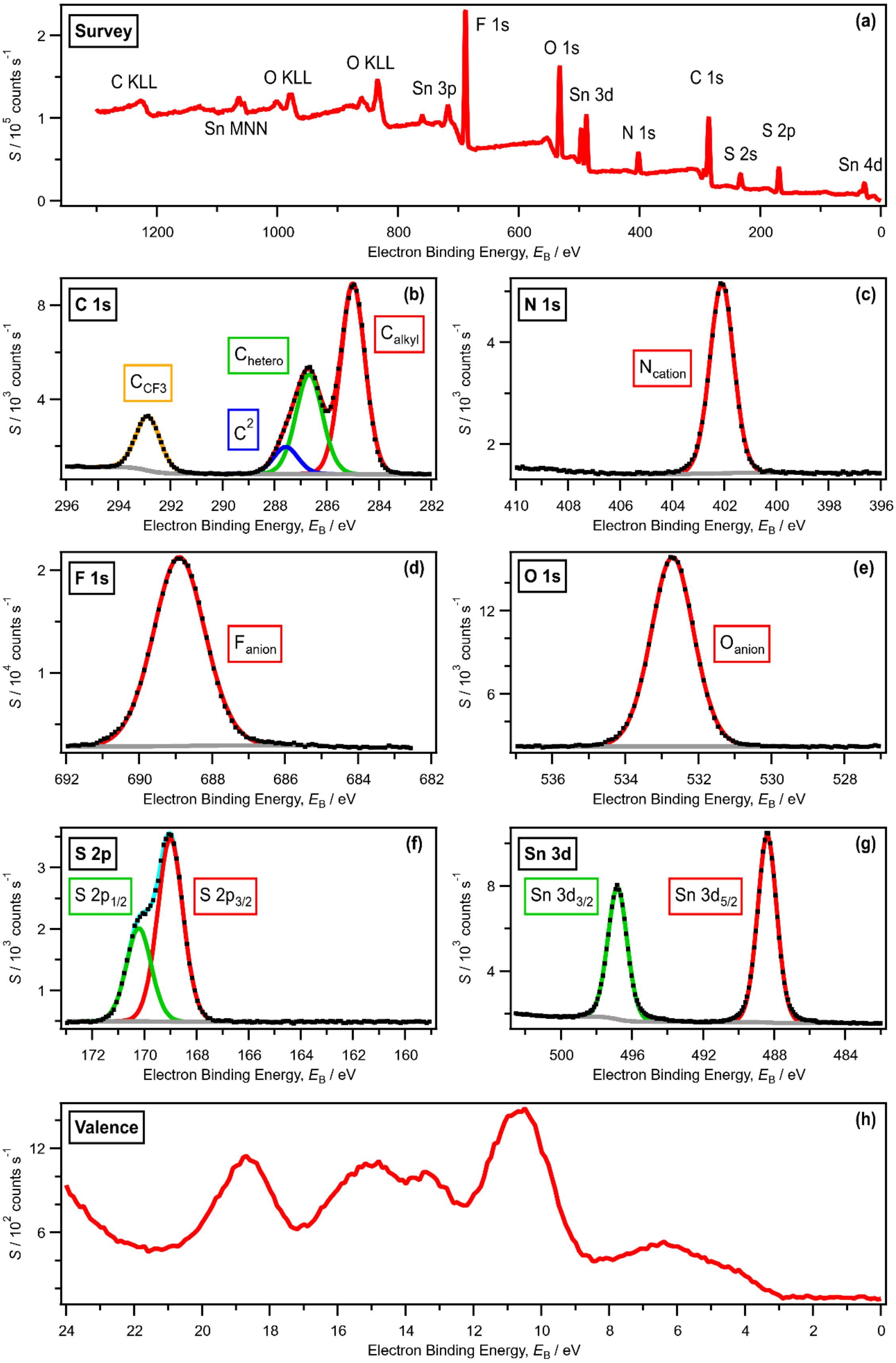

Figure S5. (a) Survey, (b-g) core and (h) valence XP spectra for $[C_8C_1Im]_2[Sn(CF_3SO_3)_3][CF_3SO_3]$ recorded on laboratory–based XPS apparatus at $h\nu = 1486.6$ eV. All electron spectra are charge referenced using procedures outlined in ESI Section 6.

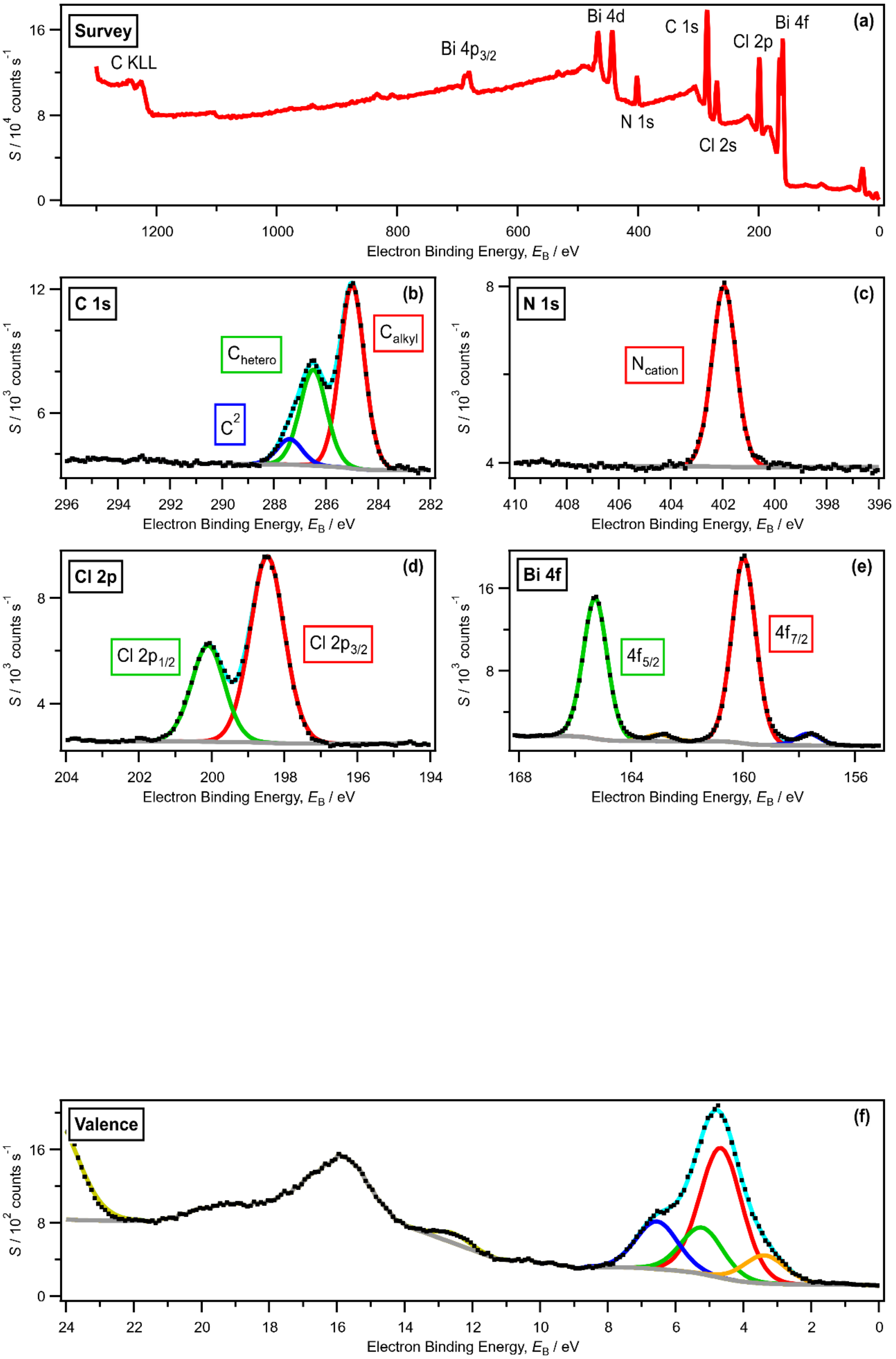

Figure S6. (a) Survey, (b-e) core and (f) valence XP spectra for $[C_8C_1Im]_2[Bi_2Cl_8]$ recorded on laboratory–based XPS apparatus at $h\nu$ = 1486.6 eV. All electron spectra are charge referenced using procedures outlined in ESI Section 6.

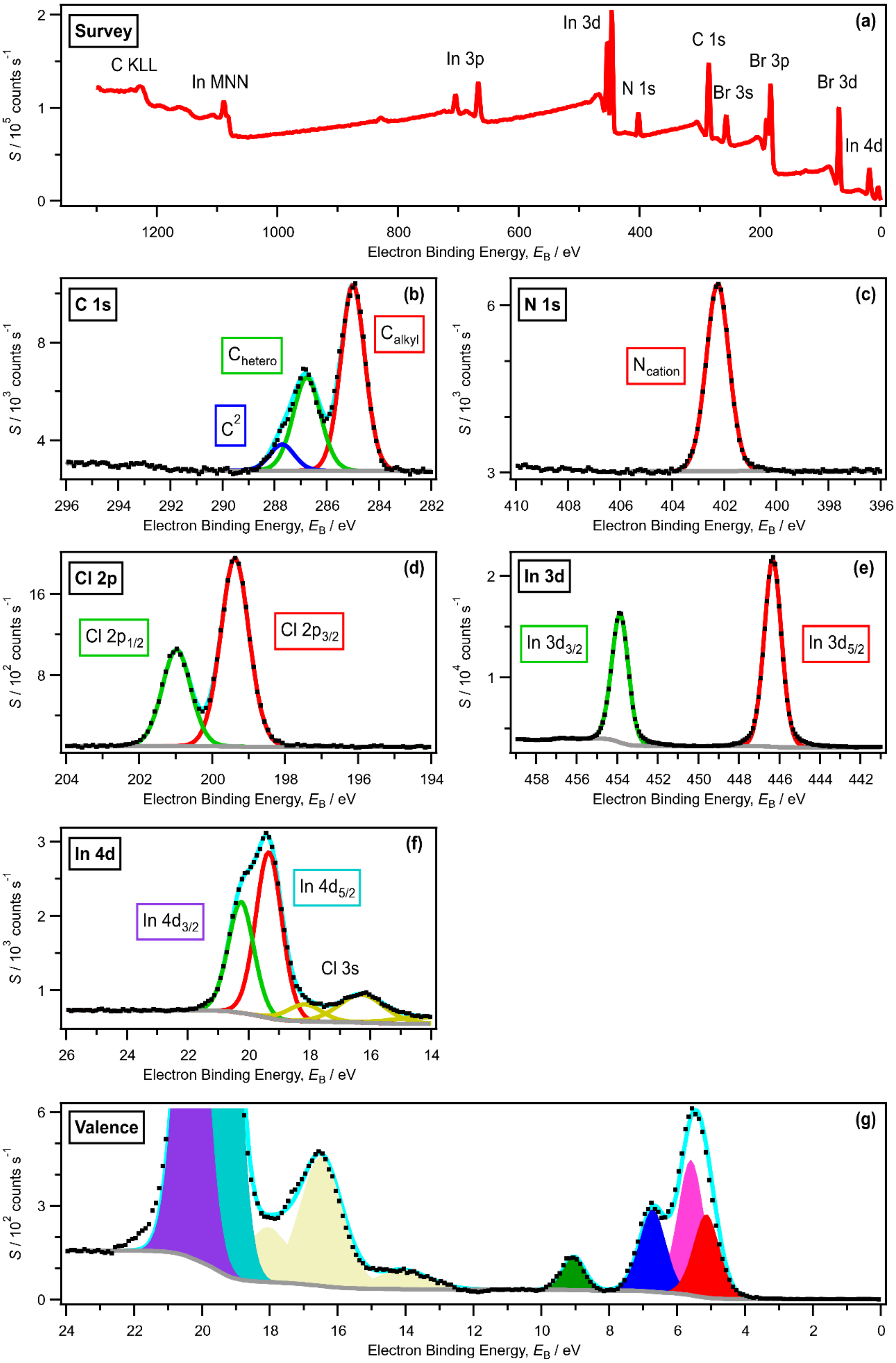

Figure S7. (a) Survey, (b-f) core and (g) valence XP spectra for $[C_8C_1Im][InBr_4]$ recorded on laboratory–based XPS apparatus at $h\nu$ = 1486.6 eV. All electron spectra are charge referenced using procedures outlined in ESI Section 6.

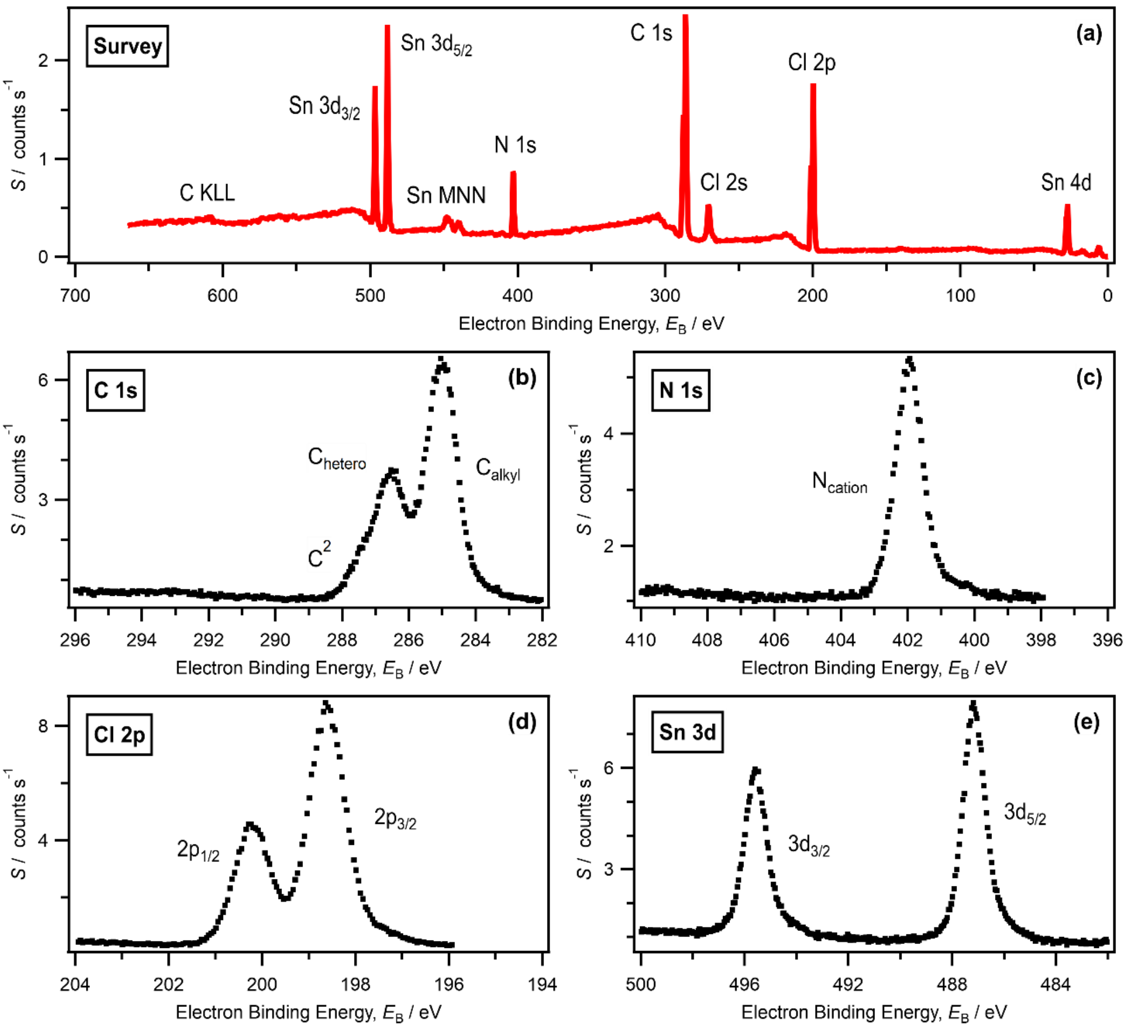

Figure S8. (a) Survey and (b-e) core XP spectra for $[C_8C_1Im][SnCl_3]$ recorded on synchrotron-based XPS apparatus at $h\nu$ = 870 eV. Electron spectra were charge referenced by -6.06 eV using $E_B(C_{alkyl}\ 1s)$ = 285.00 eV. All high-resolution XP spectra were recorded with pass energy = 50 eV.

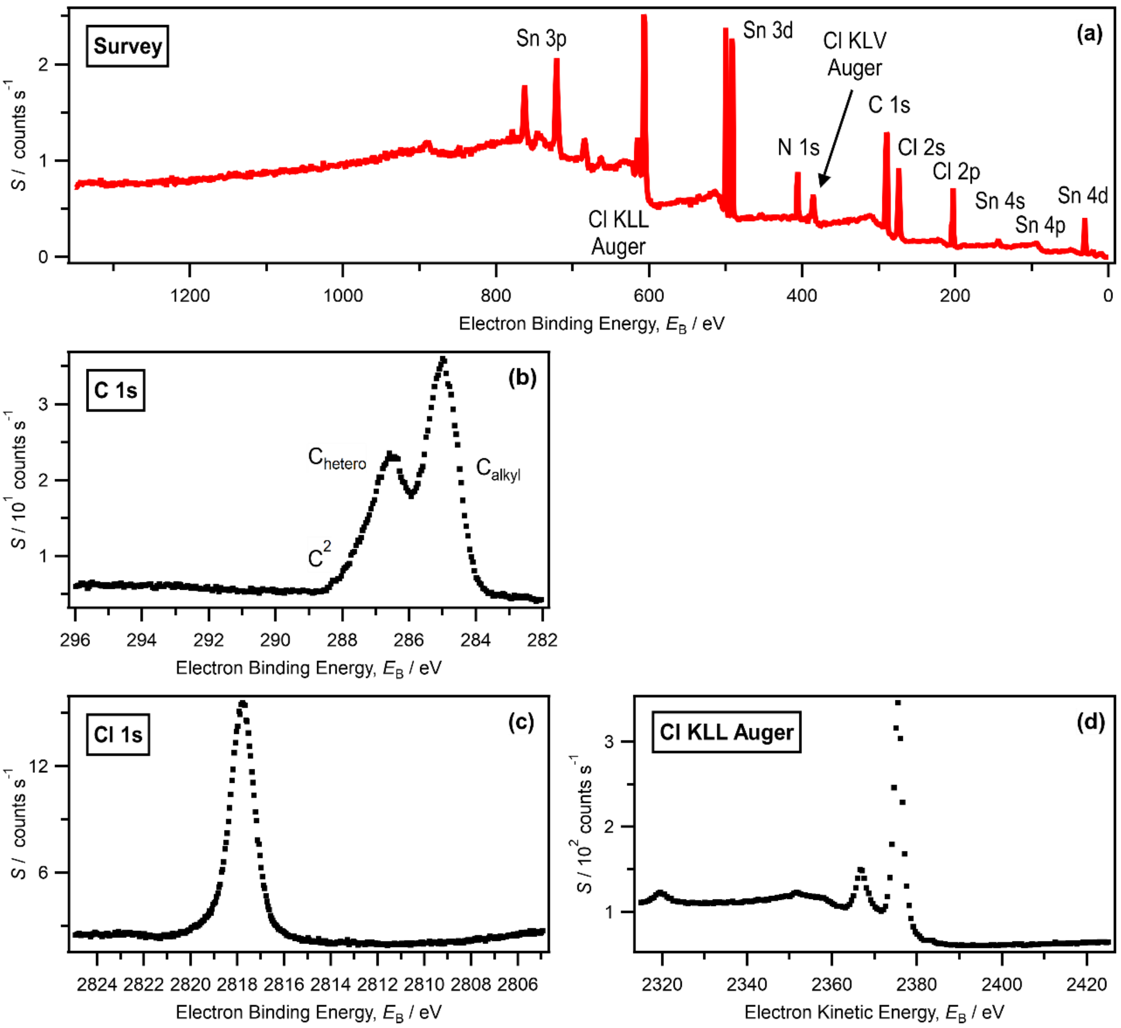

Figure S9. (a) Survey XP spectrum, (b-c) core XP spectra and Auger spectrum for $[C_8C_1Im][SnCl_3]$ recorded on synchrotron-based XPS apparatus at $h\nu$ = 2984 eV. Electron spectra were charge referenced by -5.06 eV using $E_B(C_{alkyl}\ 1s)$ = 285.00 eV. Note that the pass energies for the C 1s, Cl 1s and Cl KLL Auger were different (200 eV, 50 eV and 500 eV respectively), which has an impact on the binding energies (meaning that the binding energies for these scans are not comparable). The C 1s, Cl 1s and Cl KLL spectra were recorded with pass energy = 200 eV, 50 eV and 500 eV, respectively.

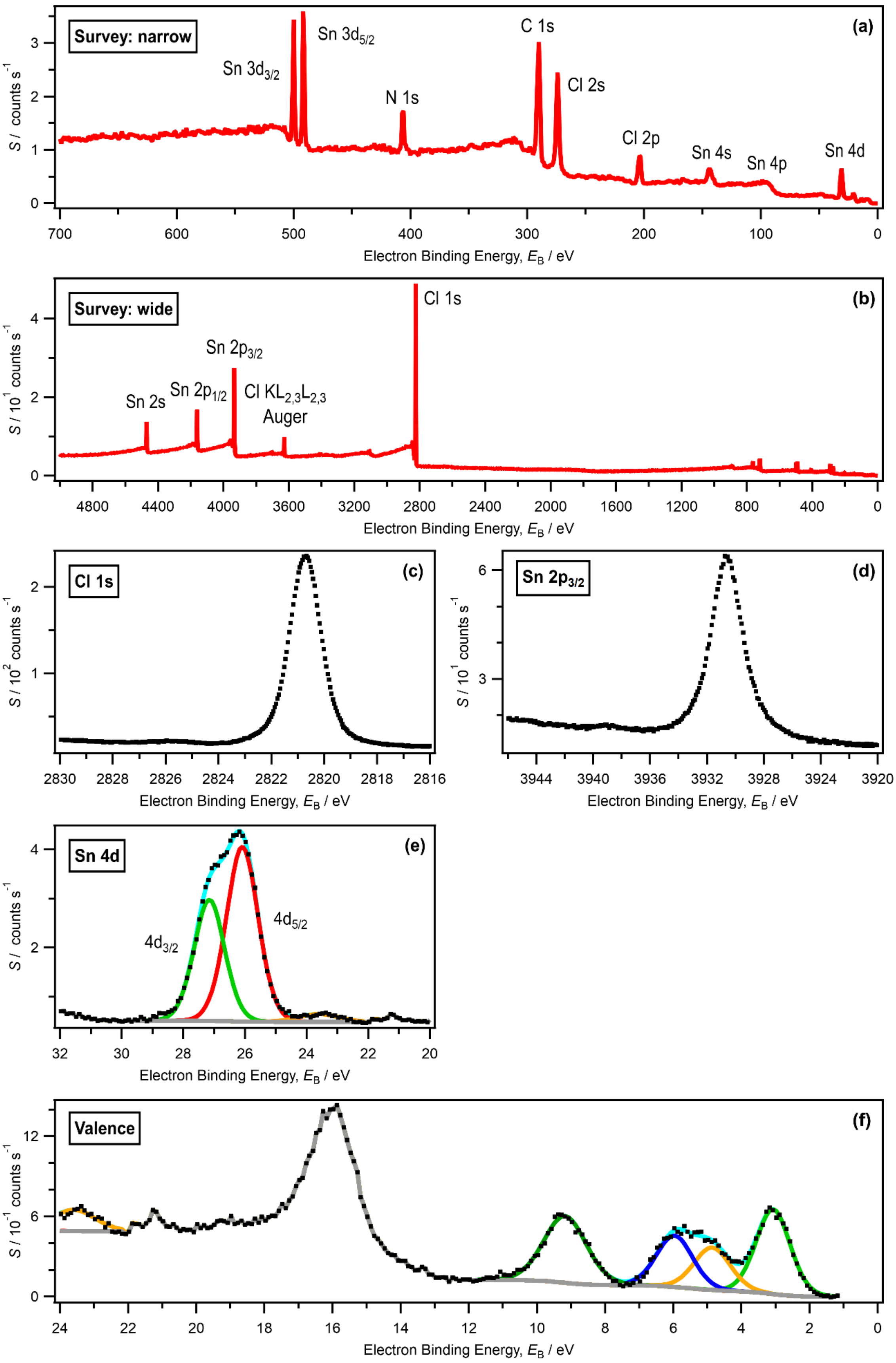

Figure S10. (a-b) Survey, (b-e) core and valence XP spectra for $[C_8C_1Im][SnCl_3]$ recorded on synchrotron-based XPS apparatus at $h\nu$ = 6000 eV. Electron spectra were charge referenced by +1.17 eV using E_B(Sn $4d_{5/2}$) = 26.09 eV for the valence XP spectrum. All high resolution XP spectra were recorded with pass energy = 200 eV.

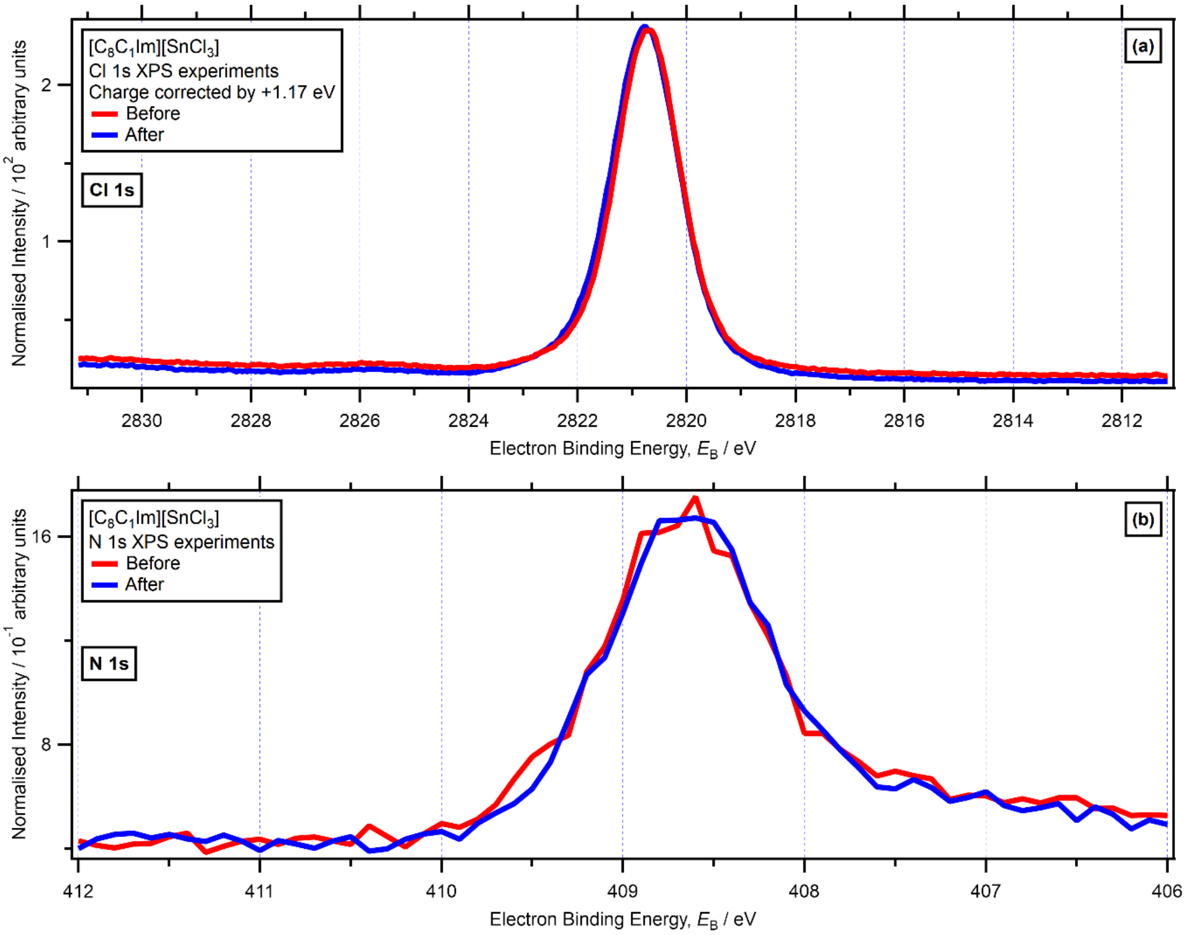

Figure S11. Demonstrating no X-ray damage over time for synchrotron valence XP spectra. (a) Cl 1s XP spectra for $[C_8C_1Im][SnCl_3]$ recorded on synchrotron-based XPS apparatus at $h\nu$ = 6000 eV, before and after valence XP spectra were recorded for $[C_8C_1Im][SnCl_3]$ at $h\nu$ = 6000 eV. Electron spectra were charge referenced by +1.17 eV using E_B(Sn $4d_{5/2}$) = 26.09 eV for the valence XP spectra. (b) N 1s XP spectra for $[C_8C_1Im][SnCl_3]$ recorded on synchrotron-based XPS apparatus at $h\nu$ = 870 eV, before and after valence XP spectra were recorded for $[C_8C_1Im][SnCl_3]$ at $h\nu$ = 250 eV. Electron spectra were not charge referenced.

Table S8. Measured experimental and nominal (in brackets) stoichiometries for the ionic liquids studied in this work, recorded at $h\nu$ = 1486.6 eV.

			C 1s	N 1s	O 1s	F 1s	S 2p	Sn $3d_{5/2}$
IL no.	Abbreviation	RSF [a]	0.205	0.350				4.300
5	$[C_8C_1Im]_2[Sn(CF_3SO_3)_3][CF_3SO_3]$	Measured (nominal)	29.9 (28)	4.0 (4)	11.2 (12)	10.6 (12)	4.4 (4)	0.9 (1)

[a] RSF = relative sensitivity factors, taken from reference [30] for C 1s, N 1s, O 1s, F 1s, S $2p_{3/2}$, and from reference [31] for Sn $3d_{5/2}$

The measured experimental and nominal stoichiometries (ESI Table S8) match well for $[C_8C_1Im]_2[Sn(CF_3SO_3)_3][CF_3SO_3]$ newly investigated here. Differences, especially the larger carbon values relative to other elements, are likely due to differences in relative sensitivity factors (RSF) values, as these RSF values were not tuned specially to the Reading XPS apparatus (in references [30, 32] RSF values were tuned specially to the XPS apparatus used in those studies). This match, along with the high-quality XP spectra given in ESI Figure S4 to ESI Figure S10, demonstrates the high purity of the IL samples newly presented here.

10. Results. XPS: Valence XPS for $[C_8C_1Im][SnCl_3]$ versus perovskites

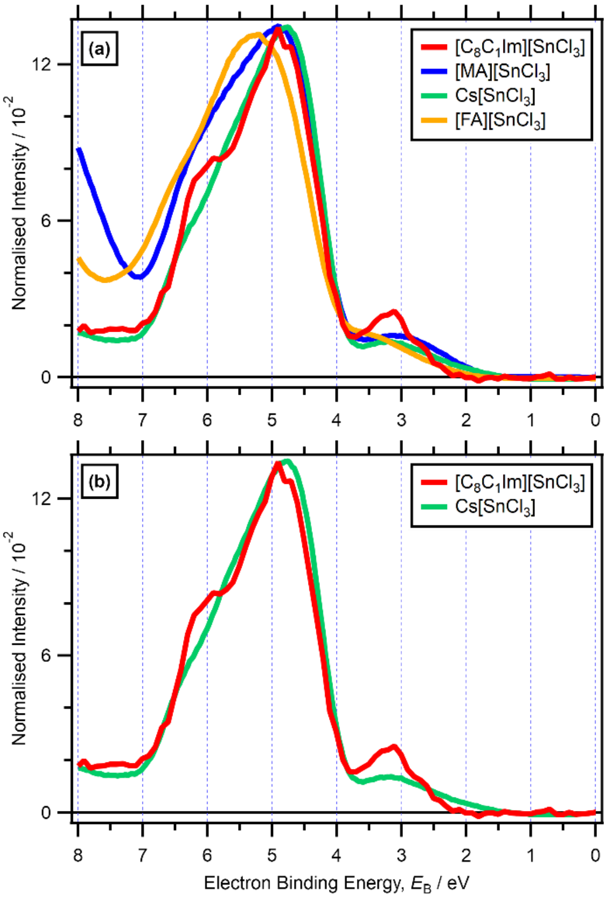

Figure S12. Data for the perovskites were from reference [33], kindly supplied by Selina Olthof (University of Cologne).

11. XPS: cation contributions to valence XPS

The focus of this paper is on anion contributions to the Density of States (DoS). Lab-based ($h\nu$ = 1486.6 eV) valence XPS spectra were dominated by anion contributions in the near-HOMO region (12 eV > E_B > 0 eV, *e.g*. Figure S13) because photoionization cross-sections for cationic AOs (*i.e*. C 2p and N 2p) are so low, relative to Cl 3p, Br 4p, and metal *n*s and *n*p AOs (ESI Section 7). Therefore, cationic contributions to these valence states can be disregarded during most analyses here. The AOs due to cationic-based MOs, *i.e*. those based upon C 2p and N 2p, are reasonably important at $h\nu$ = 250 eV (and hence are considered for the Gelius-weighted DoS, Figure S15d), but not important at all at $h\nu$ = 6000 eV (Figure S15f and Figure S2).[22, 23] Therefore, the cation HOFO are not considered here. Identification of the valence states is focused solely on states arising from the anion, *i.e*. the anion HOFO for IL XPS experiments (where cations are present, so the IL HOMO may come from either the cation or the anion, see reference [1]) and the HOMO for lone anion calculations (for lone anion calculations the HOMO is always the anion HOFO too).

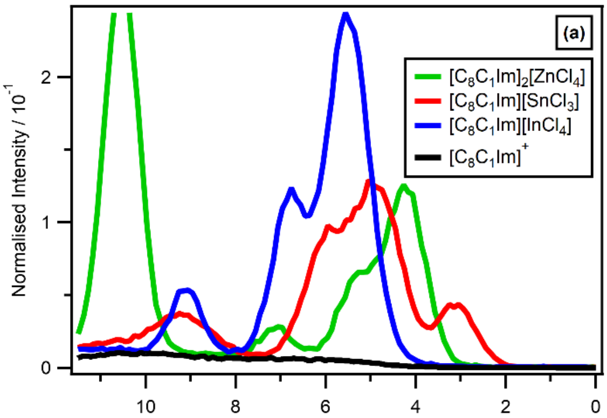

Figure S13. Area normalised valence XP spectra for $[C_8C_1Im]_2[ZnCl_4]$, $[C_8C_1Im][InCl_4]$ and $[C_8C_1Im][SnCl_3]$ at $h\nu$ = 1486.6 eV. All XP spectra were charge referenced to $E_B(C_{alkyl}\ 1s)$ = 285.0 eV. Further details on the procedures used for charge referencing of XP spectra are outlined in ESI Section 6. The $[C_8C_1Im]^+$ was taken from reference [1]. The areas were normalised as explained in reference [1].

12. Results. DFT: Atomic orbital contributions to molecular orbitals

Table S9. MO group descriptions, HOMO numbers, E_B values unshifted and shifted to match experimental data, and AO contributions to MOs for Cl^- (lone ion in an SMD)

MO group description	HOMO-	E_B / eV	E_B(shifted) / eV	Cl 3s	Cl 3p	Cl 3d
Cl 3s	3	18.3	15.8	1.00	0.00	0.00
Cl 3p	2	6.0	3.5	0.00	1.00	0.00
Cl 3p	1	6.0	3.5	0.00	1.00	0.00
Cl 3p	0	6.0	3.5	0.00	1.00	0.00

Table S10. MO group descriptions, HOMO numbers, E_B values unshifted and shifted to match experimental data, and AO contributions to MOs for $[SnCl_3]^-$ (lone ion in an SMD)

MO group description	HOMO-	E_B / eV	E_B(shifted) / eV	Sn 5s	Sn 5p	Cl 3s	Cl 3p	Cl 3d	Total Sn	Total Cl
Cl 3s + Sn 5s	12	20.7	18.2	0.06	0.01	0.93	0.00	0.00	0.07	0.93
Cl 3s	11	20.3	17.8	0.00	0.01	0.98	0.01	0.00	0.01	0.99
Cl 3s	10	20.3	17.8	0.00	0.01	0.98	0.01	0.00	0.01	0.99
Hal *np* + M *ns* bonding	9	11.9	9.5	0.64	0.01	0.04	0.31	0.00	0.65	0.35
Hal *np* + M *np* bonding	8	8.8	6.3	0.00	0.13	0.01	0.86	0.00	0.13	0.87
Hal *np* + M *np* bonding	7	8.8	6.3	0.00	0.13	0.01	0.86	0.00	0.13	0.87
Hal *np* + M *np* bonding	6	8.1	5.7	0.03	0.08	0.00	0.89	0.00	0.11	0.89
Hal *np* weakly bonding/non-bonding	5	7.8	5.3	0.00	0.00	0.00	1.00	0.00	0.00	1.00
Hal *np* weakly bonding/non-bonding	4	7.8	5.3	0.00	0.00	0.00	1.00	0.00	0.00	1.00
Hal *np* weakly anti-bonding	3	7.4	4.9	0.00	0.01	0.00	0.99	0.00	0.01	0.99
Hal *np* weakly anti-bonding	2	7.4	4.9	0.00	0.01	0.00	0.99	0.00	0.01	0.99
Hal *np* weakly anti-bonding	1	7.3	4.8	0.00	0.00	0.00	1.00	0.00	0.00	1.00
Hal *np* + M *ns* + M *np* anti-bonding	0	6.2	3.8	0.31	0.17	0.01	0.51	0.00	0.48	0.52

Table S11. MO group descriptions, HOMO numbers, E_B values unshifted and shifted to match experimental data, and AO contributions to MOs for $[ZnCl_4]^{2-}$ (lone ion in an SMD)

MO group description	HOMO-	E_B / eV	E_B(shifted) / eV	Zn 4s	Zn 3d	Zn 4p	Cl 3s	Cl 3p	Cl 3d	Total Zn	Total Cl
Cl 3s + Zn 4s	20	19.7	17.2	0.04	0.00	0.00	0.96	0.00	0.00	0.04	0.96
Cl 3s	19	19.5	17.0	0.00	0.02	0.02	0.95	0.01	0.00	0.04	0.96
Cl 3s	18	19.5	17.0	0.00	0.02	0.02	0.95	0.01	0.00	0.04	0.96
Cl 3s	17	19.5	17.0	0.00	0.02	0.02	0.95	0.01	0.00	0.04	0.96
Zn 3d	16	15.9	13.4	0.00	0.99	0.00	0.00	0.01	0.00	0.99	0.01
Zn 3d	15	15.9	13.4	0.00	0.99	0.00	0.00	0.01	0.00	0.99	0.01
Zn 3d	14	15.9	13.4	0.00	0.96	0.01	0.02	0.01	0.00	0.97	0.03
Zn 3d	13	15.9	13.4	0.00	0.96	0.01	0.02	0.01	0.00	0.97	0.03
Zn 3d	12	15.9	13.4	0.00	0.96	0.01	0.02	0.01	0.00	0.97	0.03
Hal *n*p + M *n*s bonding	11	9.1	6.6	0.30	0.00	0.00	-0.01	0.70	0.00	0.30	0.70
Hal *n*p + M *n*p bonding	10	7.5	5.1	0.00	0.00	0.09	0.00	0.90	0.00	0.10	0.90
Hal *n*p + M *n*p bonding	9	7.5	5.1	0.00	0.00	0.09	0.00	0.90	0.00	0.10	0.90
Hal *n*p + M *n*p bonding	8	7.5	5.0	0.00	0.00	0.09	0.00	0.90	0.00	0.10	0.90
Hal *n*p weakly bonding/non-bonding	7	6.9	4.4	0.00	0.01	0.00	0.00	0.99	0.00	0.01	0.99
Hal *n*p weakly bonding/non-bonding	6	6.9	4.4	0.00	0.01	0.00	0.00	0.99	0.00	0.01	0.99
Hal *n*p weakly bonding/non-bonding	5	6.7	4.2	0.00	0.01	0.03	-0.01	0.97	0.00	0.04	0.96
Hal *n*p weakly bonding/non-bonding	4	6.7	4.2	0.00	0.01	0.03	-0.01	0.97	0.00	0.04	0.96
Hal *n*p weakly bonding/non-bonding	3	6.7	4.2	0.00	0.01	0.03	-0.01	0.97	0.00	0.04	0.96
Hal *n*p weakly anti-bonding	2	6.5	4.1	0.00	0.00	0.00	0.00	1.00	0.00	0.00	1.00
Hal *n*p weakly anti-bonding	1	6.5	4.1	0.00	0.00	0.00	0.00	1.00	0.00	0.00	1.00
Hal *n*p weakly anti-bonding	0	6.5	4.1	0.00	0.00	0.00	0.00	1.00	0.00	0.00	1.00

Table S12. MO group descriptions, HOMO numbers, E_B values unshifted and shifted to match experimental data, and AO contributions to MOs for $[InCl_4]^-$ (lone ion in an SMD)

MO group description	HOMO-	E_B / eV	E_B(shifted) / eV	In 5s	In 5p	Cl 3s	Cl 3p	Cl 3d	Total In	Total Cl
Cl 3s + In 5s	15	21.4	18.9	0.09	0.00	0.91	0.00	0.00	0.09	0.91
Cl 3s	14	21.0	18.5	0.00	0.01	0.98	0.01	0.00	0.01	0.99
Cl 3s	13	21.0	18.5	0.00	0.01	0.98	0.01	0.00	0.01	0.99
Cl 3s	12	21.0	18.5	0.00	0.01	0.98	0.01	0.00	0.01	0.99
Hal *n*p + M *n*s bonding	11	11.5	9.1	0.42	0.00	0.03	0.54	0.00	0.42	0.58
Hal *n*p + M *n*p bonding	10	9.4	6.9	0.00	0.13	0.01	0.86	0.00	0.13	0.87
Hal *n*p + M *n*p bonding	9	9.4	6.9	0.00	0.13	0.01	0.86	0.00	0.13	0.87
Hal *n*p + M *n*p bonding	8	9.4	6.9	0.00	0.13	0.01	0.86	0.00	0.13	0.87
Hal *n*p weakly bonding/non-bonding	7	8.3	5.8	0.00	0.00	0.00	1.00	0.00	0.00	1.00
Hal *n*p weakly bonding/non-bonding	6	8.2	5.8	0.00	0.00	0.00	1.00	0.00	0.00	1.00
Hal *n*p weakly bonding/non-bonding	5	8.1	5.7	0.00	0.03	-0.01	0.98	0.00	0.03	0.97
Hal *n*p weakly bonding/non-bonding	4	8.1	5.6	0.00	0.03	-0.01	0.98	0.00	0.03	0.97
Hal *n*p weakly bonding/non-bonding	3	8.1	5.6	0.00	0.03	-0.01	0.98	0.00	0.03	0.97
Hal *n*p weakly anti-bonding	2	7.9	5.4	0.00	0.00	0.00	1.00	0.00	0.00	1.00
Hal *n*p weakly anti-bonding	1	7.9	5.4	0.00	0.00	0.00	1.00	0.00	0.00	1.00
Hal *n*p weakly anti-bonding	0	7.9	5.4	0.00	0.00	0.00	1.00	0.00	0.00	1.00

Table S13. MO group descriptions, HOMO numbers, E_B values unshifted and shifted to match experimental data, and AO contributions to MOs for $[Bi_2Cl_8]^{2-}$ (lone ion in an SMD)

MO group description	HOMO-	E_B / eV	E_B(shifted) / eV	Bi 6s	Bi 6p	Cl 3s	Cl 3p	Cl 3d	Total Bi	Total Cl
Bi 5d	43	30.1	27.6	0.00	0.00	0.00	0.00	0.00	0.99	0.01
Bi 5d	42	30.1	27.6	0.00	0.00	0.00	0.00	0.00	0.99	0.01
Bi 5d	41	30.0	27.6	0.00	0.00	0.01	0.00	0.00	0.99	0.01
Bi 5d	40	30.0	27.6	0.00	0.00	0.00	0.00	0.00	1.00	0.00
Bi 5d	39	30.0	27.5	0.00	0.00	0.00	0.00	0.00	1.00	0.00
Bi 5d	38	30.0	27.5	0.00	0.00	0.00	0.00	0.00	1.00	0.00
Bi 5d	37	30.0	27.5	0.00	0.00	0.00	0.00	0.00	1.00	0.00
Bi 5d	36	30.0	27.5	0.00	0.00	0.00	0.00	0.00	1.00	0.00
Bi 5d	35	30.0	27.5	0.00	0.00	0.00	0.00	0.00	1.00	0.00
Bi 5d	34	30.0	27.5	0.00	0.00	0.00	0.00	0.00	1.00	0.00
Cl 3s + Bi 6s	33	21.2	18.8	0.10	0.00	0.88	0.02	0.00	0.09	0.91
Cl 3s + Bi 6s	32	21.1	18.6	0.07	-0.01	0.91	0.02	0.00	0.06	0.94
Cl 3s	31	20.5	18.0	0.01	0.00	1.00	0.00	0.00	0.00	1.00
Cl 3s	30	20.4	17.9	0.00	0.00	1.00	0.00	0.00	0.00	1.00
Cl 3s	29	20.4	17.9	0.00	0.00	1.00	0.00	0.00	0.00	1.00
Cl 3s	28	20.4	17.9	0.00	-0.01	1.00	0.00	0.00	0.00	1.00
Cl 3s	27	20.2	17.7	0.00	0.00	1.01	0.00	0.00	-0.01	1.01
Cl 3s	26	20.2	17.7	0.00	0.00	1.02	0.00	0.00	-0.02	1.02
Hal *n*p + M *n*s bonding	25	16.0	13.6	0.73	0.00	0.13	0.12	0.01	0.73	0.27
Hal *n*p + M *n*s bonding	24	15.8	13.3	0.72	0.00	0.16	0.11	0.01	0.72	0.28
Hal *n*p + M *n*p bonding	23	9.6	7.1	0.00	0.21	0.01	0.77	0.01	0.22	0.78
Hal *n*p + M *n*p bonding	22	9.5	7.0	0.00	0.21	0.02	0.77	0.01	0.21	0.79
Hal *n*p + M *n*p bonding	21	9.3	6.8	0.01	0.20	0.01	0.76	0.01	0.22	0.78
Hal *n*p + M *n*p bonding	20	9.3	6.8	0.00	0.19	0.01	0.79	0.01	0.20	0.80
Hal *n*p + M *n*p bonding	19	9.2	6.7	0.01	0.18	0.00	0.79	0.01	0.20	0.80
Hal *n*p + M *n*p bonding	18	9.0	6.6	0.00	0.19	0.01	0.79	0.01	0.19	0.81
Hal *n*p weakly bonding/non-bonding/ weakly anti-bonding	17	8.1	5.6	0.00	0.00	0.00	0.98	0.00	0.02	0.98
Hal *n*p weakly bonding/non-bonding/ weakly anti-bonding	16	8.0	5.5	0.00	0.00	0.00	0.98	0.00	0.02	0.98
Hal *n*p weakly bonding/non-bonding/ weakly anti-bonding	15	7.9	5.4	0.00	0.00	0.00	0.99	0.00	0.01	0.99
Hal *n*p weakly bonding/non-bonding/ weakly anti-bonding	14	7.8	5.4	0.00	0.00	0.00	0.99	0.00	0.01	0.99
Hal *n*p weakly bonding/non-bonding/ weakly anti-bonding	13	7.8	5.3	0.00	0.00	0.00	0.98	0.00	0.03	0.97
Hal *n*p weakly bonding/non-bonding/ weakly anti-bonding	12	7.8	5.3	0.00	0.01	0.00	0.97	0.00	0.03	0.97

Hal *n*p weakly bonding/non-bonding/ weakly anti-bonding	11	7.7	5.2	0.00	0.00	-0.01	0.99	0.00	0.02	0.98
Hal *n*p weakly bonding/non-bonding/ weakly anti-bonding	10	7.6	5.1	0.00	0.01	-0.01	1.00	0.00	0.01	0.99
Hal *n*p weakly bonding/non-bonding/ weakly anti-bonding	9	7.5	5.1	0.01	0.00	-0.01	0.99	0.00	0.01	0.99
Hal *n*p weakly bonding/non-bonding/ weakly anti-bonding	8	7.5	5.0	0.00	0.00	0.00	1.00	0.00	0.00	1.00
Hal *n*p weakly bonding/non-bonding/ weakly anti-bonding	7	7.4	4.9	0.00	0.01	-0.01	0.99	0.00	0.01	0.99
Hal *n*p weakly bonding/non-bonding/ weakly anti-bonding	6	7.4	4.9	0.00	0.00	0.00	1.00	0.00	0.00	1.00
Hal *n*p weakly bonding/non-bonding/ weakly anti-bonding	5	7.4	4.9	0.00	0.00	0.00	1.00	0.00	0.00	1.00
Hal *n*p weakly bonding/non-bonding/ weakly anti-bonding	4	7.3	4.9	0.00	0.00	0.00	1.00	0.00	0.00	1.00
Hal *n*p weakly bonding/non-bonding/ weakly anti-bonding	3	7.3	4.8	0.00	0.00	0.00	1.00	0.00	0.00	1.00
Hal *n*p weakly bonding/non-bonding/ weakly anti-bonding	2	7.2	4.7	0.00	0.00	0.00	1.00	0.00	0.00	1.00
Hal *n*p + M *n*s + M *n*p anti-bonding	1	6.8	4.3	0.08	0.02	0.00	0.88	0.00	0.12	0.88
Hal *n*p + M *n*s + M *n*p anti-bonding	0	6.7	4.2	0.09	0.02	0.00	0.88	0.00	0.12	0.88

13. Results. XPS + DFT: variable $h\nu$ valence XPS data for $[C_8C_1Im][SnCl_3]$

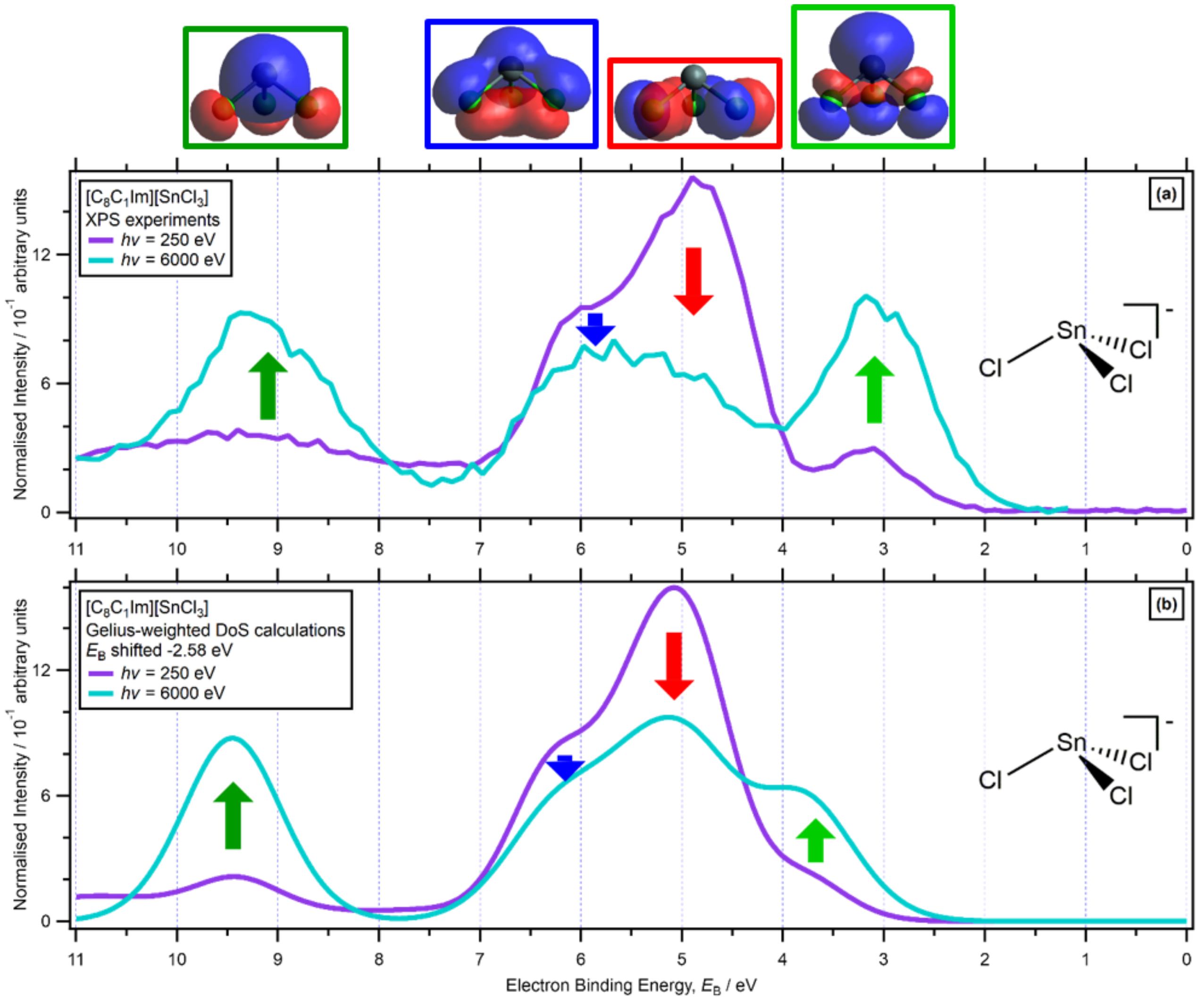

Figure S14. (a) Area normalised experimental valence XP spectra for $[C_8C_1Im][SnCl_3]$ at $h\nu$ = 250 eV and $h\nu$ = 6000 eV. (b) Area normalised Gelius-weighted DoS calculations for a sum of $[C_8C_1Im]^+$ (lone ion in an SMD) and $[SnCl_3]^-$ (lone ion in an SMD) at $h\nu$ = 250 eV (FWHM = 0.8 eV) and $[SnCl_3]^-$ (lone ion in an SMD) at $h\nu$ = 6000 eV (FWHM = 1.3 eV). Representative MOs are given for each group of valence states; the arrow colours match the box colours. The areas were normalised using procedures outlined in ESI Section 8. All XP spectra were charge referenced to $E_B(C_{alkyl}$ 1s) = 285.00 eV. The calculated E_B were shifted -2.48 eV, to give the best visual match to the experimental data. Further details on the procedures used for charge referencing of XP spectra are outlined in ESI Section 6.

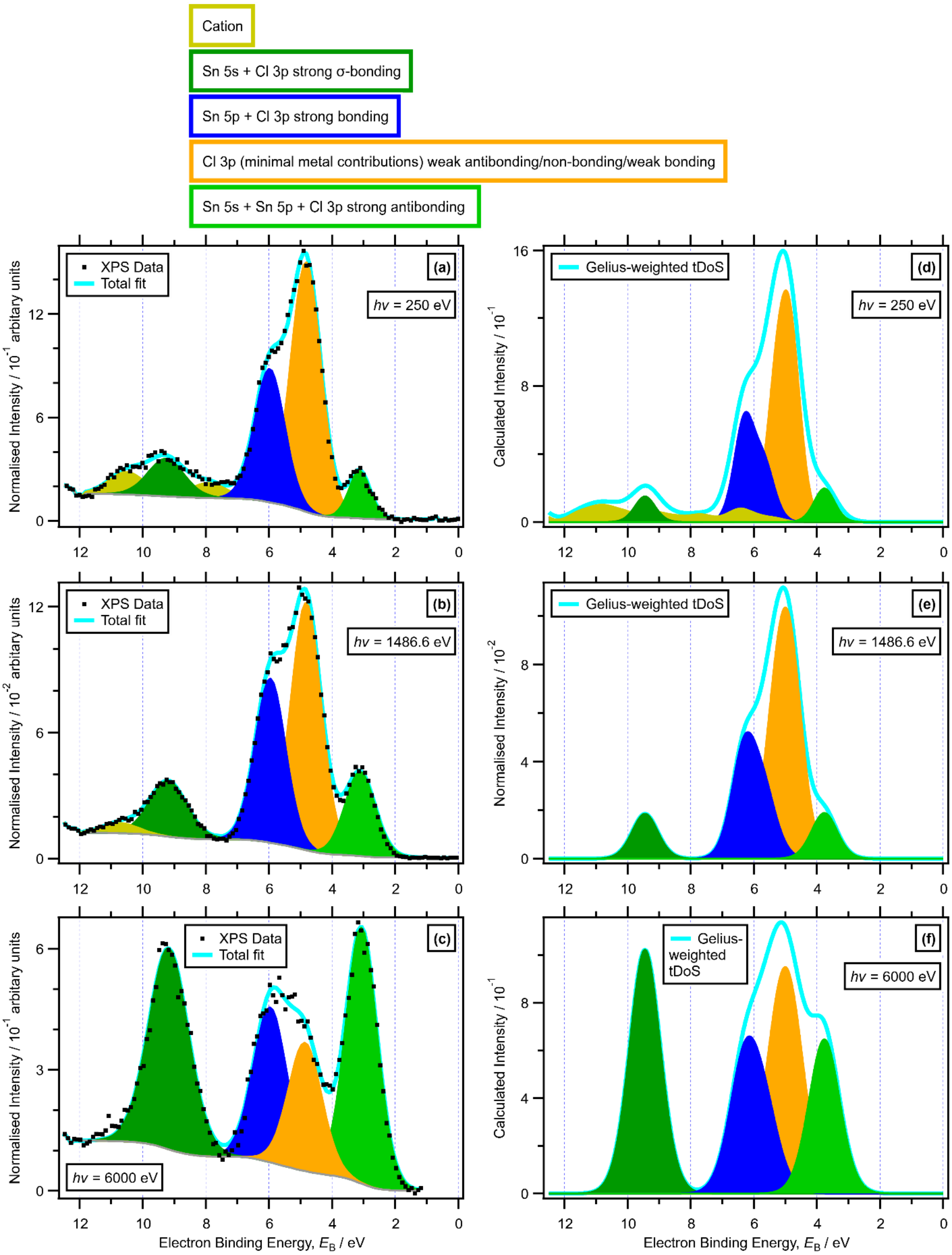

Figure S15. Visual comparison of experimental XP spectra and Gelius-weighted DoS for $[C_8C_1Im][SnCl_3]$; one component was fitted for Cl 3p weakly anti-bonding + Cl 3p weakly bonding/non-bonding. Area normalised valence XP spectra at: (a) $h\nu$ = 250 eV, (b) $h\nu$ = 1486.6 eV and (c) $h\nu$ = 6000 eV. Gelius-weighted DoS for lone metal complex anions in an IL SMD for: (d) $[SnCl_3]^-$ (FWHM = 0.8 eV) + $[C_8C_1Im]^+$ (FWHM = 1.0 eV) at $h\nu$ = 250 eV, (e) $[SnCl_3]^-$ (FWHM = 1.0 eV) at $h\nu$ = 1486.6 eV and (f) $[SnCl_3]^-$ (FWHM = 1.3 eV) at $h\nu$ = 6000 eV. A single linear shift of -2.48 eV was applied to the Gelius-weighted DoS to give the best overall visual match of the experimental XP spectra and Gelius-weighted DoS. For the Gelius-weighted DoS calculated at $h\nu$ = 250 eV and at $h\nu$ = 6000 eV, the asymmetry parameter was accounted for, as explained in ESI Section 3.2.4. All XP

spectra were charge referenced to $E_B(C_{alkyl}\ 1s)$ = 285.00 eV. Further details on the procedures used for charge referencing of XP spectra are outlined in ESI Section 6. The areas were normalised using procedures outlined in reference [1].

Table S14. XPS data for $[C_8C_1Im][SnCl_3]$. XP spectra measured at $h\nu$ = 1486.6 eV are charge referenced to $E_B(C_{alkyl}\ 1s)$ = 285.00 eV. All other XP spectra are charge referenced to $E_B(Sn\ 4d_{5/2})$ = 26.09 eV. Further details on the procedures used for charge referencing of XP spectra are outlined in ESI Section 6. E_B(average) and E_B(standard deviation) were determined using data recorded at three different $h\nu$ ($h\nu$ = 250 eV, $h\nu$ = 1486.6 eV, $h\nu$ = 6000 eV). The component area ratios were determined for component area (for $h\nu$ = 6000 eV) / component area (for $h\nu$ = 250 eV). The normalised component area ratios were determined by setting the component area ratio = 1 for the component due to Cl 3p weakly bonding/non-bonding/weakly anti-bonding.

	Sn $4d_{3/2}$	Sn $4d_{5/2}$	Component ii [a]	Cl 3p + Sn 5s bonding	Component i [a]	Cl 3p + Sn 5p bonding	Cl 3p weakly bonding/non-bonding/weakly anti-bonding [b]	Cl 3p + Sn 5s + Sn 5p anti-bonding
E_B (for $h\nu$ = 1486.6 eV) / eV	27.16	26.09	10.70	9.19		5.95	4.81	3.09
E_B (for $h\nu$ = 250 eV) / eV	27.16	26.09	10.50	9.26	7.91	5.98	4.82	3.13
E_B (for $h\nu$ = 6000 eV) / eV	27.16	26.09		9.18		5.94	4.79	3.05
E_B(average) / eV	27.16	26.09		9.21		5.96	4.81	3.09
E_B(standard deviation) / eV				0.05		0.02	0.02	0.04
FWHM (for $h\nu$ = 1486.6 eV) / eV	0.97	1.00	1.28	1.38		1.16	1.16	0.99
FWHM (for $h\nu$ = 250 eV) / eV	0.86	0.81	1.35	1.35	1.35	1.12	1.12	0.79
FWHM (for $h\nu$ = 6000 eV) / eV	1.30	1.34		1.72		1.47	1.47	1.35
Component area (for $h\nu$ = 1486.6 eV) / arbitrary units	2046	3069	21	122		297	449	140
Component area (for $h\nu$ = 250 eV) / arbitrary units	96	144	19	32	13	94	175	24
Component area (for $h\nu$ = 6000 eV) / arbitrary units	493	739		133		92	68	126
Component area ratio ($h\nu$ = 6000 eV to $h\nu$ = 250 eV)	5.1	5.1		4.2		1.0	0.4	5.3
Normalised component area ratio ($h\nu$ = 6000 eV to $h\nu$ = 250 eV)	13.4	13.4		10.8		2.5	1.0	13.7

[a] Component i and component ii were due to the cation; these components were only present at lower $h\nu$, due to photoionization cross-sections

[b] Cl 3p weakly bonding/non-bonding/weakly anti-bonding fitted with one component here for consistency across XP spectra recorded at all $h\nu$

14. Results. XPS versus Gelius-weighted DoS

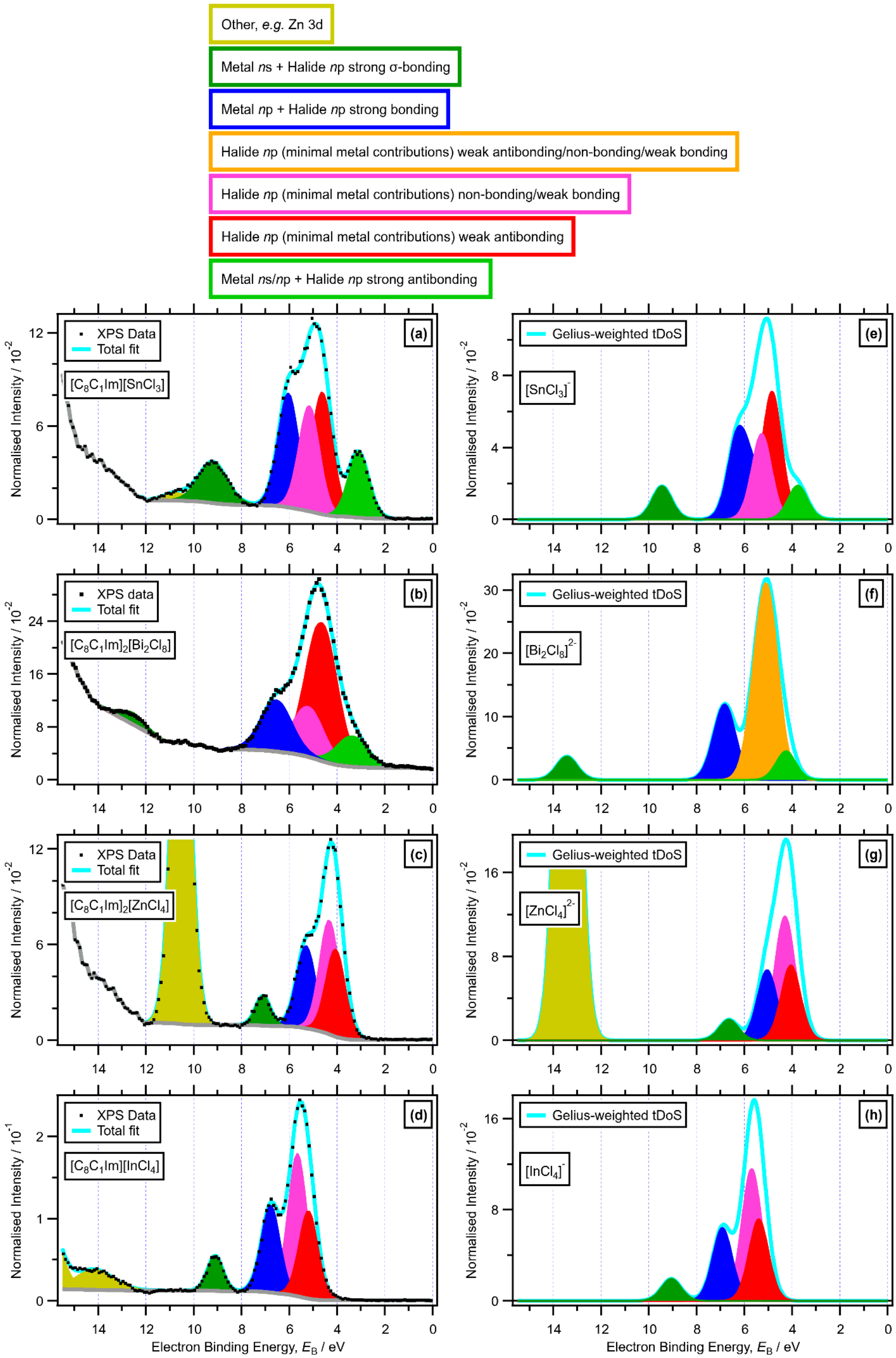

Other, e.g. Zn 3d
Metal ns + Halide np strong σ-bonding
Metal np + Halide np strong bonding
Halide np (minimal metal contributions) weak antibonding/non-bonding/weak bonding
Halide np (minimal metal contributions) non-bonding/weak bonding
Halide np (minimal metal contributions) weak antibonding
Metal ns/np + Halide np strong antibonding
(a) XPS Data, Total fit, $[C_8C_1Im][SnCl_3]$
(b) XPS data, Total fit, $[C_8C_1Im]_2[Bi_2Cl_8]$
(c) XPS Data, Total fit, $[C_8C_1Im]_2[ZnCl_4]$
(d) XPS Data, Total fit, $[C_8C_1Im][InCl_4]$
(e) Gelius-weighted tDoS, $[SnCl_3]^-$
(f) Gelius-weighted tDoS, $[Bi_2Cl_8]^{2-}$
(g) Gelius-weighted tDoS, $[ZnCl_4]^{2-}$
(h) Gelius-weighted tDoS, $[InCl_4]^-$
Normalised Intensity / 10^{-2}
Normalised Intensity / 10^{-1}
Electron Binding Energy, E_B / eV

Figure S16. Visual comparison of experimental XP spectra and Gelius-weighted DoS. Area normalised valence XP spectra at $h\nu$ = 1486.6 eV for: (a) $[C_8C_1Im][SnCl_3]$, (b) $[C_8C_1Im]_2[Bi_2Cl_8]$, (c) $[C_8C_1Im]_2[ZnCl_4]$ and (d) $[C_8C_1Im][InCl_4]$. All XP spectra were charge referenced to $E_B(C_{alkyl}$ 1s) = 285.00 eV. Further details on the procedures used for charge referencing of XP spectra are outlined in ESI Section 6. Gelius-weighted DoS (at $h\nu$ = 1486.6 eV) for lone metal complex anions in an IL SMD for: (e) $[SnCl_3]^-$ (FWHM = 1.0 eV), (f) $[Bi_2Cl_8]^{2-}$ (FWHM = 1.0 eV), (g) $[ZnCl_4]^{2-}$ (FWHM = 1.0 eV) and (h) $[InCl_4]^-$ (FWHM = 1.0 eV). A single linear shift of -2.48 eV was applied to the Gelius-weighted DoS to give the best overall visual match of the experimental XP spectra and Gelius-weighted DoS. The XP spectra areas were normalised using procedures outlined in ESI Section 8.

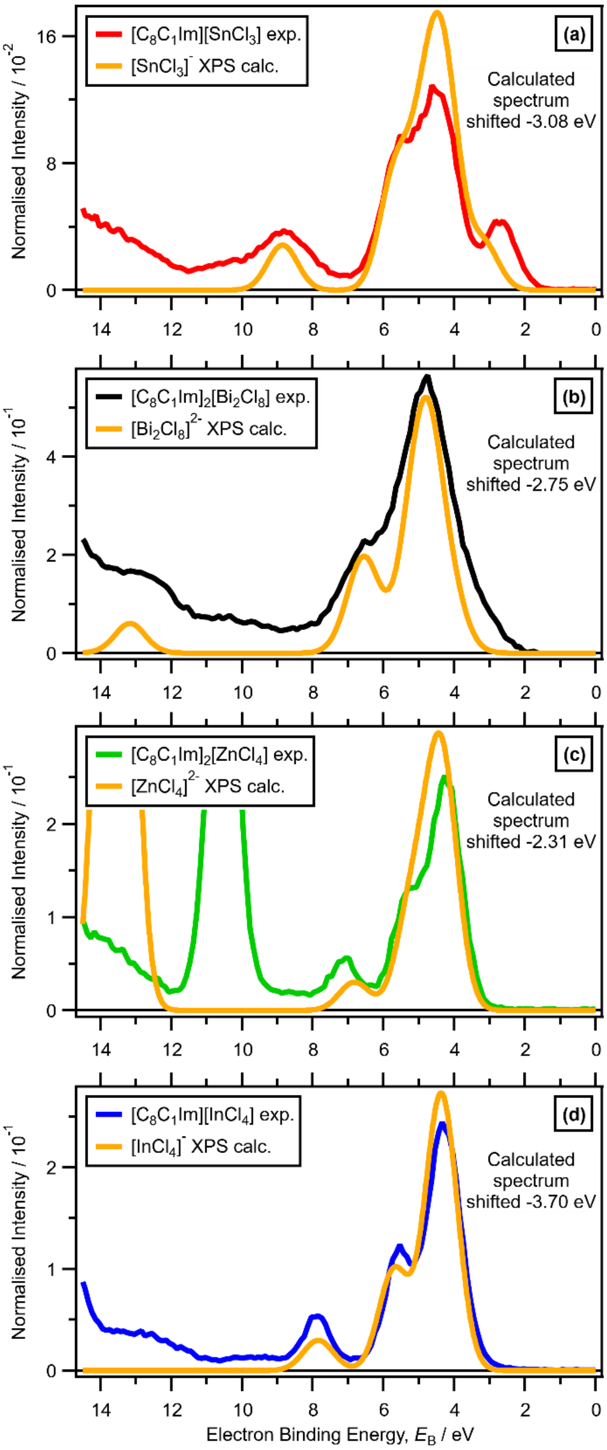

Figure S17. Visual comparison of experimental XP spectra and Gelius-weighted DoS. Area normalised valence XP spectra at $h\nu$ = 1486.6 eV and Gelius-weighted DoS (at $h\nu$ = 1486.6 eV) for lone metal complex anions in an IL SMD (FWHM = 1.0 eV) for: (a) $[C_8C_1Im][SnCl_3]$ and $[SnCl_3]^-$, (b) $[C_8C_1Im]_2[Bi_2Cl_8]$ and $[Bi_2Cl_8]^{2-}$, (c) $[C_8C_1Im]_2[ZnCl_4]$ and $[ZnCl_4]^{2-}$, (d) $[C_8C_1Im][InCl_4]$ and $[InCl_4]^-$. All XP spectra were charge referenced to $E_B(C_{alkyl}\ 1s)$ = 285.00 eV. Further details on the procedures used for charge referencing of XP spectra are outlined in ESI Section 6. Linear shifts are applied to the Gelius-weighted DoS to give the best overall visual match of the experimental XP spectra and Gelius-weighted DoS. The XP spectra areas were normalised using procedures outlined in ESI Section 8.

15. Results. DFT: tDoS, pDoS and MO representations

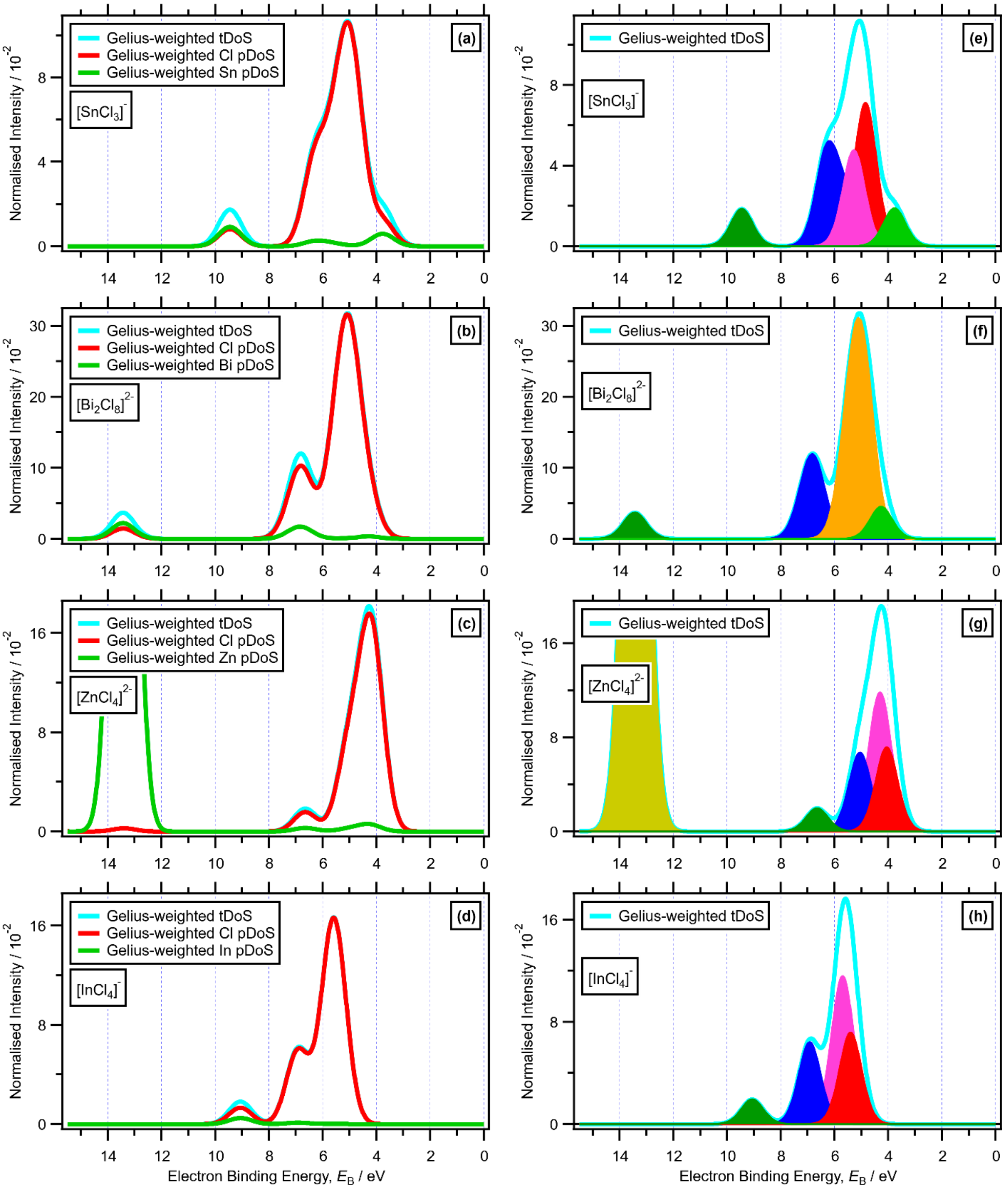

Figure S18. Lone ion (SMD) DFT calculations to give DoS and atomic pDoS contributions for: (a) $[SnCl_3]^-$, (b) $[Bi_2Cl_8]^{2-}$, (c) $[ZnCl_4]^{2-}$ and (d) $[InCl_4]^-$. Lone ion (SMD) DFT calculations to give tDoS and MO groups (see Table S15): (e) $[SnCl_3]^-$, (f) $[Bi_2Cl_8]^{2-}$, (g) $[ZnCl_4]^{2-}$ and (h) $[InCl_4]^-$. FWHM = 1.0 eV with GL(30) lineshapes were used. E_B were shifted by -2.48 eV to give good visual matches to the halide *n*p regions for the experimental XP spectra (charge referenced for $E_B(C_{alkyl}\ 1s)$ = 285.00 eV).

Table S15. Comparing XPS experiments of pDoS calculations. Grouping occupied MOs for lone anion SMD for $[ZnCl_4]^{2-}$, $[InCl_4]^{-}$, $[SnCl_3]^{-}$ and $[Bi_2Cl_8]^{2-}$. Representative MOs are given for each grouping of MOs. E_B(exp.) = experimental binding energies, charge referenced to E_B(C_{alkyl} 1s) = 285.00 eV. Further details on the procedures used for charge referencing of XP spectra are outlined in ESI Section 6. E_B(calc.) = calculated binding energies, shifted by -2.48 eV, so that good visual matches were obtained between the experimental XP spectra and Gelius-weighted DoS.

Component Identity	Representative MOs for $[ZnCl_4]^{2-}$	E_B(exp.) for $[C_8C_1Im]_2[ZnCl_4]$ / eV	E_B(calc.) for $[ZnCl_4]^{2-}$ / eV	Representative MOs for $[InCl_4]^{-}$	E_B(exp.) for $[C_8C_1Im][InCl_4]$ / eV	E_B(calc.) for $[InCl_4]^{-}$ / eV	Representative MOs for $[SnCl_3]^{-}$	E_B(exp.) for $[C_8C_1Im][SnCl_3]$ / eV	E_B(calc.) for $[SnCl_3]^{-}$ / eV	Representative MOs for $[Bi_2Cl_8]^{2-}$ [a]	E_B(exp.) for $[C_8C_1Im]_2[Bi_2Cl_8]$ / eV [a]	E_B(calc.) for $[Bi_2Cl_8]^{2-}$ / eV [a]
Hal *np* + M *ns* bonding	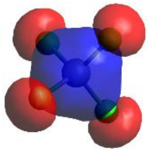 HOMO-11	7.1	HOMO-11 = 6.6	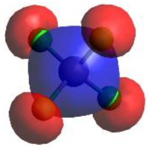 HOMO-11	9.1	HOMO-11 = 9.1	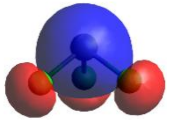 HOMO-9	9.2	HOMO-9 = 9.5	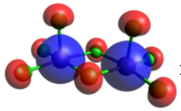 HOMO-24 [a]	12.6	HOMO-25 = 13.6<br>HOMO-24 = 13.3
Hal *np* + M *np* bonding	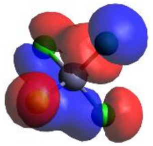 HOMO-8	5.3	HOMO-10 = 5.1<br>HOMO-9 = 5.1<br>HOMO-8 = 5.0	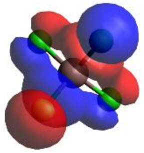 HOMO-8	6.8	HOMO-10 = 6.9<br>HOMO-9 = 6.9<br>HOMO-8 =6.9	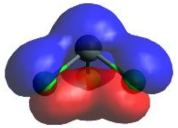 HOMO-6	6.0	HOMO-8 = 6.3<br>HOMO-7 = 6.3<br>HOMO-6 = 5.7	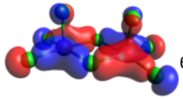 HOMO-23	6.6	HOMO-23 = 7.1<br>HOMO-22 = 7.0<br>HOMO-21 = 6.8<br>HOMO-20 = 6.8<br>HOMO-19 = 6.7<br>HOMO-18 = 6.6
Hal *np* weakly bonding/non-bonding	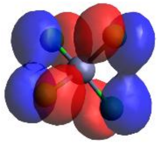 HOMO-7<br>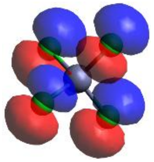 HOMO-3	4.3	HOMO-7 = 4.4<br>HOMO-6 = 4.4<br>HOMO-5 = 4.2<br>HOMO-4 = 4.2<br>HOMO-3 =4.2	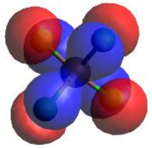 HOMO-7<br>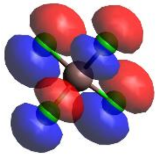 HOMO-3	5.7	HOMO-7 = 5.8<br>HOMO-6 = 5.8<br>HOMO-5 = 5.7<br>HOMO-4 = 5.6<br>HOMO-3 =5.6	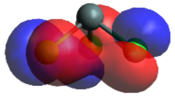 HOMO-5	5.2	HOMO-5 = 5.3<br>HOMO-4 = 5.3	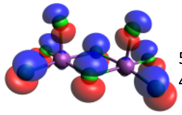 HOMO-10	5.2<br>4.7	HOMO-17 = 5.6<br>HOMO-16 = 5.5<br>HOMO-15 = 5.4<br>HOMO-14 = 5.4<br>HOMO-13 = 5.3<br>HOMO-12 = 5.3<br>HOMO-11 = 5.2<br>HOMO-10 = 5.1<br>HOMO-9 = 5.1<br>HOMO-8 = 5.0<br>HOMO-7 = 4.9<br>HOMO-6 = 4.9<br>HOMO-5 = 4.9<br>HOMO-4 = 4.9<br>HOMO-3 = 4.8<br>HOMO-2 = 4.7
Hal *np* weakly anti-bonding	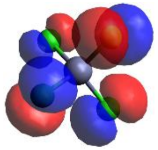	4.1	HOMO-2 = 4.1<br>HOMO-1 = 4.1<br>HOMO = 4.1	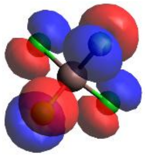	5.2	HOMO-2 = 5.4<br>HOMO-1 = 5.4<br>HOMO = 5.4	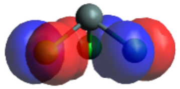 HOMO-1	4.6	HOMO-3 = 4.9<br>HOMO-2 = 4.9<br>HOMO-1 = 4.8			

	HOMO	HOMO						
Hal *n*p + M *n*s + M *n*p anti-bonding			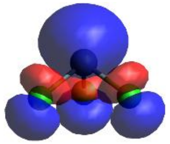<br>HOMO	3.1	HOMO = 3.8	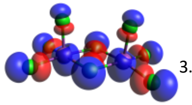<br>HOMO	3.4	HOMO-1 = 4.3<br>HOMO = 4.2

[a] The MOs that gave rise to the component labelled as Hal *n*p + M *n*s bonding for $[Bi_2Cl_8]^{2-}$) had significant Cl 3s contributions, as well as Cl 3p + Bi 6s contributions

16. Results. XPS: valence XPS for $[Sn(CF_3SO_3)_3]^-$ versus $[CF_3SO_3]^-$

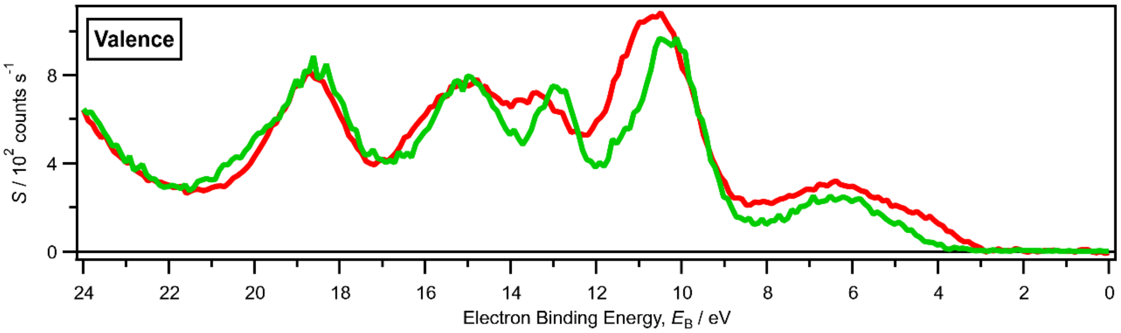

Figure S19. Valence XPS for $[C_8C_1Im]_2[Sn(CF_3SO_3)_3][CF_3SO_3]$ and $[C_8C_1Im][CF_3SO_3]$. Area normalisation is by eye. All electron spectra are charge referenced using procedures outlined in ESI Section 6.

17. Results. XPS: core XPS for $[Sn(CF_3SO_3)_3]^-$ versus $[SnBr_3]^-$ and $[SnBr_3]^-$

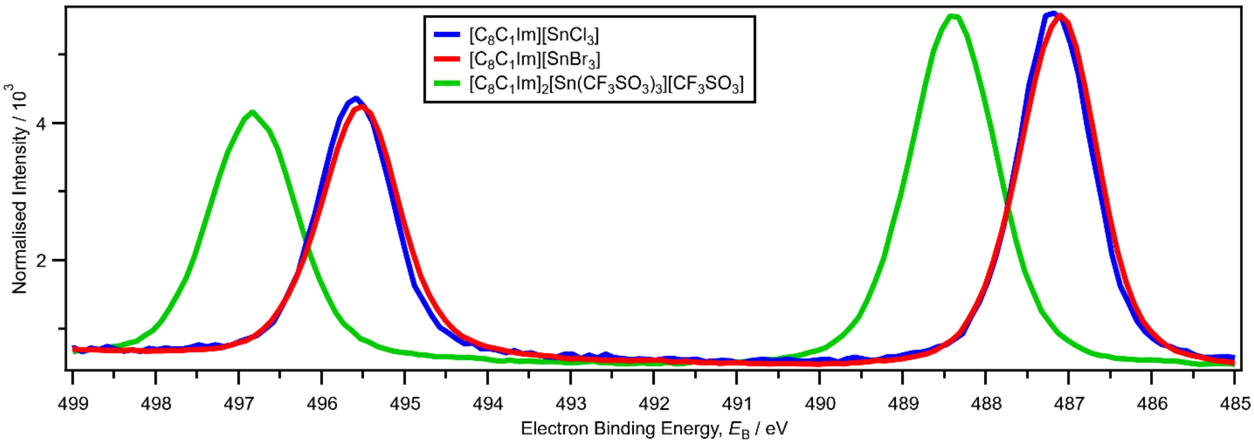

Figure S20. Core Sn 3d XPS for $[C_8C_1Im]_2[Sn(CF_3SO_3)_3][CF_3SO_3]$, $[C_8C_1Im][SnCl_3]$ and $[C_8C_1Im][SnBr_3]$. Area normalisation is by eye. All electron spectra are charge referenced using procedures outlined in ESI Section 6.

18. Results. XPS: E_B(cation core) vs. E_B(anion valence) comparisons

Table S16. E_B(N_{cation} 1s), E_B(Cl 3p), E_B(Br 4p) used for correlation plots. All for E_B recorded at $h\nu$ = 1486.6 eV. For E_B recorded at $h\nu$ = 250 eV and $h\nu$ = 6000 eV for $[C_8C_1Im][SnCl_3]$, the values were the same, within error (ESI Table S14). Further details on the procedures used for charge referencing of XP spectra are outlined in ESI Section 6.

IL no.	IL	E_B(N_{cation} 1s) / eV from reference [2]	E_i = E_B(HOMO) / eV	E_B(Cl 3p) / eV	E_B(Br 4p) / eV
1	$[C_8C_1Im]Cl$	401.68	3.48	3.48	
2	$[C_8C_1Im]Br$	401.71	3.07		3.07
7	$[C_8C_1Im]_2[ZnCl_4]$	401.82	4.06	4.06	
8	$[C_8C_1Im]_2[ZnBr_4]$	401.88	3.48		3.48
6	$[C_8C_1Im]_2[Bi_2Cl_8]$	401.95	4.66	4.66	
9	$[C_8C_1Im]_2[Zn_2Br_6]$	401.96	3.80		3.80
10	$[C_8C_1Im]_2[Zn_2Cl_6]$	401.97	4.39	4.39	
4	$[C_8C_1Im][SnBr_3]$	401.99	2.67		3.90
3	$[C_8C_1Im][SnCl_3]$	402.00	3.10	4.62	
11	$[C_8C_1Im]_2[Zn_3Br_8]$	402.03	4.11		4.11
12	$[C_8C_1Im]_2[Zn_4Br_{10}]$	402.06	4.18		4.18
13	$[C_8C_1Im]_2[Zn_4Cl_{10}]$	402.06	5.00	5.00	
14	$[C_8C_1Im][InCl_4]$	402.19	5.20	5.20	
15	$[C_8C_1Im][InBr_4]$	402.26	4.51		4.51

19. Results. DFT: Atomic charges

Table S17. Mulliken calculated atomic charges for a lone $[SnCl_3]^-$ ion in the SMD.

Element	Mulliken charge / e
Sn	0.80
Cl	-0.60
Cl	-0.60
Cl	-0.60

Table S18. Mulliken calculated atomic charges for a lone $[ZnCl_4]^{2-}$ ion in the SMD.

Element	Mulliken charge / e
Zn	0.40
Cl	-0.60
Cl	-0.60
Cl	-0.60
Cl	-0.60

Table S19. Mulliken calculated atomic charges for a lone $[InCl_4]^-$ ion in the SMD.

Element	Mulliken charge / e
In	0.97
Cl	-0.49
Cl	-0.49
Cl	-0.49
Cl	-0.49

Table S20. Mulliken calculated atomic charges for a lone $[Bi_2Cl_8]^{2-}$ ion in the SMD.

Element	Mulliken charge / e
Bi	1.80
Bi	1.80
Cl	-0.72
Cl	-0.71
Cl	-0.71
Cl	-0.72
Cl	-0.72
Cl	-0.72
Cl	-0.64

20. Results. DFT: Coordinates

Table S21. Coordinates for a lone $[SnCl_3]^-$ ion in the SMD.

Element	*x*	*y*	*z*
Sn	0.000741	0.002933	0.695573
Cl	-1.831651	1.120504	-0.683080
Cl	1.891072	1.015434	-0.684511
Cl	-0.061601	-2.144565	-0.678211

Table S22. Coordinates for a lone $[ZnCl_4]^{2-}$ ion in the SMD.

Element	*x*	*y*	*z*
Zn	-0.002361	0.000965	-0.001170
Cl	1.124359	-0.520108	2.033203
Cl	-2.358582	-0.009756	0.364064
Cl	0.557055	-1.624773	-1.645906
Cl	0.681335	2.152934	-0.749296

Table S23. Coordinates for a lone $[InCl_4]^-$ ion in the SMD.

Element	*x*	*y*	*z*
In	-0.196998	0.834897	0.000000
Cl	0.549681	1.890833	1.828953
Cl	-2.436998	0.834924	0.000000
Cl	0.549643	-1.277005	0.000000
Cl	0.549681	1.890833	-1.828953

Table S24. Coordinates for a lone $[Bi_2Cl_8]^{2-}$ ion in the SMD.

Element	*x*	*y*	*z*
Bi	2.17049	0.00371	-0.22824
Bi	-2.17052	-0.00369	-0.22808
Cl	3.96899	-1.90675	-0.56833
Cl	-2.29950	0.07764	2.31540
Cl	0.00406	-1.92395	-0.21027
Cl	-0.00409	1.92397	-0.21009
Cl	2.29972	-0.07790	2.31521
Cl	3.93624	1.96014	-0.42308
Cl	-3.93629	-1.96009	-0.42302
Cl	-3.96900	1.90683	-0.56788

21. References

(1) Fogarty, R. M.; Palgrave, R. G.; Bourne, R. A.; Handrup, K.; Villar-Garcia, I. J.; Payne, D. J.; Hunt, P. A.; Lovelock, K. R. J. Electron spectroscopy of ionic liquids: experimental identification of atomic orbital contributions to valence electronic structure. *Phys. Chem. Chem. Phys.* **2019**, *21* (35), 18893-18910.

(2) Gousseva, E.; Towers Tompkins, F. K.; Seymour, J. M.; Parker, L. G.; Clarke, C. J.; Palgrave, R. G.; Bennett, R. A.; Grau-Crespo, R.; Lovelock, K. R. J. Anion-Dependent Strength Scale of Interactions in Ionic Liquids from X-ray Photoelectron Spectroscopy, Ab Initio Molecular Dynamics, and Density Functional Theory. *J. Phys. Chem. B* **2024**, *128* (20), 5030-5043.

(3) Seymour, J. M.; Gousseva, E.; Large, A. I.; Clarke, C. J.; Licence, P.; Fogarty, R. M.; Duncan, D. A.; Ferrer, P.; Venturini, F.; Bennett, R. A.; et al. Experimental measurement and prediction of ionic liquid ionisation energies. *Phys. Chem. Chem. Phys.* **2021**, *23* (37), 20957-20973.

(4) Lee, T. L.; Duncan, D. A. A Two-Color Beamline for Electron Spectroscopies at Diamond Light Source. *Synchrotron Radiat. News* **2018**, *31* (4), 16-22.

(5) Becke, A. D. DENSITY-FUNCTIONAL EXCHANGE-ENERGY APPROXIMATION WITH CORRECT ASYMPTOTIC-BEHAVIOR. *Phys. Rev. A* **1988**, *38* (6), 3098-3100.

(6) Lee, C. T.; Yang, W. T.; Parr, R. G. DEVELOPMENT OF THE COLLE-SALVETTI CORRELATION-ENERGY FORMULA INTO A FUNCTIONAL OF THE ELECTRON-DENSITY. *Phys. Rev. B* **1988**, *37* (2), 785-789.

(7) M. J. Frisch, G. W. Trucks, H. B. Schlegel, G. E. Scuseria, M. A. Robb, J. R. Cheeseman, G. Scalmani, V. Barone, B. Mennucci, G. A. Petersson, H. Nakatsuji, M. Caricato, X. Li, H. P. Hratchian, A. F. Izmaylov, J. Bloino, G. Zheng, J. L. Sonnenberg, M. Hada, M. Ehara, K. Toyota, R. Fukuda, J. Hasegawa, M. Ishida, T. Nakajima, Y. Honda, O. Kitao, H. Nakai, T. Vreven, J. J. A. Montgomery, J. E. Peralta, F. Ogliaro, M. Bearpark, J. J. Heyd, E. Brothers, K. N. Kudin, V. N. Staroverov, R. Kobayashi, J. Normand, K. Raghavachari, A. Rendell, J. C. Burant, S. S. Iyengar, J. Tomasi, M. Cossi, N. Rega, J. M. Millam, M. Klene, J. E. Knox, J. B. Cross, V. Bakken, C. Adamo, J. Jaramillo, R. Gomperts, R. E. Stratmann, O. Yazyev, A. J. Austin, R. Cammi, C. Pomelli, J. W. Ochterski, R. L. Martin, K. Morokuma, V. G. Zakrzewski, G. A. Voth, P. Salvador, J. J. Dannenberg, S. Dapprich, A. D. Daniels, Ö. Farkas, J. B. Foresman, J. V. Ortiz, J. Cioslowski and D. J. Fox, Gaussian 09, Revision D.01 ed., Gaussian Inc., Wallingford CT, **2009**.

(8) M. J. Frisch, G. W. Trucks, H. B. Schlegel, G. E. Scuseria, M. A. Robb, J. R. Cheeseman, G. Scalmani, V. Barone, G. A. Petersson, H. Nakatsuji, X. Li, M. Caricato, A. V. Marenich, J. Bloino, B. G. Janesko, R. Gomperts, B. Mennucci, H. P. Hratchian, J. V. Ortiz, A. F. Izmaylov, J. L. Sonnenberg, D. Williams-Young, F. Ding, F. Lipparini, F. Egidi, J. Goings, B. Peng, A. Petrone, T. Henderson, D. Ranasinghe, V. G. Zakrzewski, J. Gao, N. Rega, G. Zheng, W. Liang, M. Hada, M. Ehara, K. Toyota, R. Fukuda, J. Hasegawa, M. Ishida, T. Nakajima, Y. Honda, O. Kitao, H. Nakai, T. Vreven, K. Throssell, J. J. A. Montgomery, J. E. Peralta, F. Ogliaro, M. J. Bearpark, J. J. Heyd, E. N. Brothers, K. N. Kudin, V. N. Staroverov, T. A. Keith, R. Kobayashi, J. Normand, K. Raghavachari, A. P. Rendell, J. C. Burant, S. S. Iyengar, J. Tomasi, M. Cossi, J. M. Millam, M. Klene, C. Adamo, R. Cammi, J. W. Ochterski, R. L. Martin, K. Morokuma, O. Farkas, J. B. Foresman and D. J. Fox, Gaussian 16, Revision C.01, Gaussian, Inc., Wallingford CT, **2016**.

(9) Grimme, S.; Ehrlich, S.; Goerigk, L. Effect of the Damping Function in Dispersion Corrected Density Functional Theory. *J. Comput. Chem.* **2011**, *32* (7), 1456-1465.

(10) Becke, A. D.; Johnson, E. R. A density-functional model of the dispersion interaction. *J. Chem. Phys.* **2005**, *123* (15), 154101.

(11) Becke, A. D.; Johnson, E. R. Exchange-hole dipole moment and the dispersion interaction: High-order dispersion coefficients. *J. Chem. Phys.* **2006**, *124* (1), 014104.

(12) Grimme, S.; Antony, J.; Ehrlich, S.; Krieg, H. A consistent and accurate ab initio parametrization of density functional dispersion correction (DFT-D) for the 94 elements H-Pu. *J. Chem. Phys.* **2010**, *132* (15), 154104.

(13) Rowe, R.; Lovelock, K. R. J.; Hunt, P. A. Bi(III) halometallate ionic liquids: Interactions and speciation. *J. Chem. Phys.* **2021**, *155* (1), 014501.

(14) Peterson, K. A. Systematically convergent basis sets with relativistic pseudopotentials. I. Correlation consistent basis sets for the post-d group 13-15 elements. *J. Chem. Phys.* **2003**, *119* (21), 11099-11112.

(15) Peterson, K. A.; Figgen, D.; Goll, E.; Stoll, H.; Dolg, M. Systematically convergent basis sets with relativistic pseudopotentials. II. Small-core pseudopotentials and correlation consistent basis sets for the post-d group 16-18 elements. *J. Chem. Phys.* **2003**, *119* (21), 11113-11123.

(16) Bernales, V. S.; Marenich, A. V.; Contreras, R.; Cramer, C. J.; Truhlar, D. G. Quantum Mechanical Continuum Solvation Models for Ionic Liquids. *J. Phys. Chem. B* **2012**, *116* (30), 9122-9129.

(17) Rowe, R. Halometallate Ionic Liquids and Deep Eutectic Solvents. Imperial College London, 2019.

(18) Mulliken, R. S. *J. Chem. Phys.* **1955**, *23*, 1833-1840.

(19) Lu, T.; Chen, F. W. Multiwfn: A multifunctional wavefunction analyzer. *J. Comput. Chem.* **2012**, *33* (5), 580-592.

(20) Gelius, U.; Siegbahn, K. ESCA STUDIES OF MOLECULAR CORE AND VALENCE LEVELS IN GAS-PHASE. *Faraday Discuss.* **1972**, *54*, 257-268.

(21) Gelius, U. RECENT PROGRESS IN ESCA STUDIES OF GASES. *J. Electron. Spectrosc. Relat. Phenom.* **1974**, *5* (NOV-D), 985-1057.

(22) Yeh, J. J.; Lindau, I. ATOMIC SUBSHELL PHOTOIONIZATION CROSS-SECTIONS AND ASYMMETRY PARAMETERS - 1 LESS-THAN-OR-EQUAL-TO Z LESS-THAN-OR-EQUAL-TO 103. *At. Data Nucl. Data Tables* **1985**, *32* (1), 1-155.

(23) Scofield, J. H. *Theoretical photoionization cross sections from 1 to 1500 keV*; UCRL--51326; Lawrence Livermore Laboratory, 1973. DOI: 10.2172/4545040.

(24) Briggs, D.; Grant, J. T. Surface Analysis by Auger and X-ray Photoelectron Spectroscopy. IM Publications: Manchester, 2003.
(25) Thürmer, S.; Seidel, R.; Faubel, M.; Eberhardt, W.; Hemminger, J. C.; Bradforth, S. E.; Winter, B. Photoelectron Angular Distributions from Liquid Water: Effects of Electron Scattering. *Phys. Rev. Lett.* **2013**, *111* (17), 173005.
(26) Mudd, J. J.; Lee, T. L.; Munoz-Sanjose, V.; Zuniga-Perez, J.; Payne, D. J.; Egdell, R. G.; McConville, C. F. Valence-band orbital character of CdO: A synchrotron-radiation photoelectron spectroscopy and density functional theory study. *Phys. Rev. B* **2014**, *89* (16), 165305.
(27) Rahm, M.; Zeng, T.; Hoffmann, R. Electronegativity Seen as the Ground-State Average Valence Electron Binding Energy. *J. Am. Chem. Soc.* **2019**, *141* (1), 342-351.
(28) Lovelock, K. R. J.; Villar-Garcia, I. J.; Maier, F.; Steinrück, H. P.; Licence, P. Photoelectron Spectroscopy of Ionic Liquid-Based Interfaces. *Chem. Rev.* **2010**, *110* (9), 5158-5190.
(29) Villar-Garcia, I. J.; Fearn, S.; De Gregorio, G. F.; Ismail, N. L.; Gschwend, F. J. V.; McIntosh, A. J. S.; Lovelock, K. R. J. The ionic liquid-vacuum outer atomic surface: a low-energy ion scattering study. *Chem. Sci.* **2014**, *5* (11), 4404-4418.
(30) Kolbeck, C.; Cremer, T.; Lovelock, K. R. J.; Paape, N.; Schulz, P. S.; Wasserscheid, P.; Maier, F.; Steinrück, H. P. Influence of Different Anions on the Surface Composition of Ionic Liquids Studied Using ARXPS. *J. Phys. Chem. B* **2009**, *113* (25), 8682-8688.
(31) Wagner, C. D.; Davis, L. E.; Zeller, M. V.; Taylor, J. A.; Raymond, R. H.; Gale, L. H. Empirical Atomic Sensitivity Factors for Quantitative-Analysis by Electron-Spectroscopy for Chemical-Analysis. *Surf. Interface Anal.* **1981**, *3* (5), 211-225.
(32) Kolbeck, C.; Killian, M.; Maier, F.; Paape, N.; Wasserscheid, P.; Steinrück, H. P. Surface characterization of functionalized imidazolium-based ionic liquids. *Langmuir* **2008**, *24* (17), 9500-9507.
(33) Tao, S. X.; Schmidt, I.; Brocks, G.; Jiang, J. K.; Tranca, I.; Meerholz, K.; Olthof, S. Absolute energy level positions in tin-and lead-based halide perovskites. *Nat. Commun.* **2019**, *10*, 2560.
